# The Knowledge We Hold: Exploring Occupational Therapy's Epistemic Identity and the Balance Between Evidence and Practice

**DOI:** 10.1177/00084174251383259

**Published:** 2025-10-29

**Authors:** Aliki Thomas

**Keywords:** Epistemic communities, Epistemic humility, Policy influence, Pluralism, Systems transformation

## Abstract

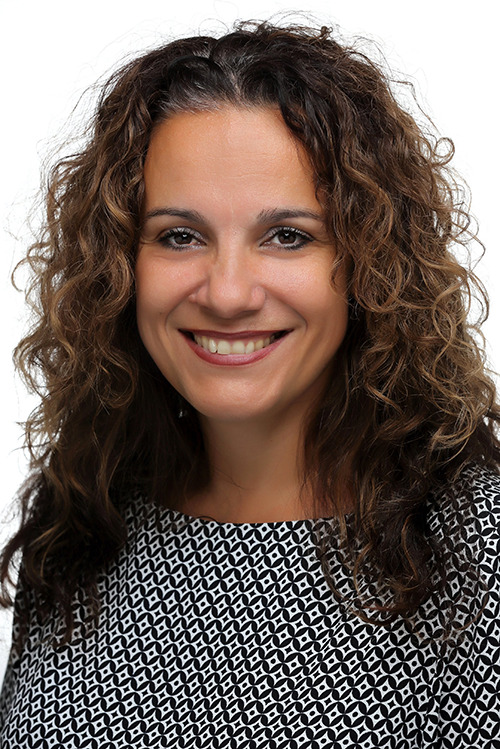
Aliki Thomas

Occupational therapy has long drawn strength from its ability to integrate diverse forms of knowledge, scientific evidence, clinical expertise, patient narratives, and embodied wisdom. This lectureship explores the profession's epistemic identity: how we develop, validate, and apply knowledge in practice, and how this identity enables us to respond to complex health and social challenges. Drawing on historical and contemporary case examples, the author examines the evolution of occupational therapy knowledge, tracing shifts from intuitive, relational care to evidence-based models and toward today's epistemically pluralistic landscape. She argues that occupational therapy is not only shaped by multiple forms of knowledge but also functions as an epistemic community, one that holds authoritative, policy-relevant knowledge and contributes meaningfully to interdisciplinary networks. This identity positions occupational therapists to influence care models, policy directions, and systems transformation. However, our influence depends on epistemic humility, strategic collaboration, and a commitment to bridging research, practice, and lived experience. The author then outlines three key calls to action: (1) strengthening our knowledge base through pluralism and reflexivity; (2) amplifying our collective voice by clarifying professional identity and reducing fragmentation; and (3) increasing our policy influence through purposeful collaboration and leadership. In rapidly evolving healthcare environments, occupational therapy must assert its role not only in delivering services, but in stewarding integrated, context-sensitive knowledge that drives equity, participation, and justice.

## Introduction

From a young age, I felt a deep calling to help others—to address injustice and advocate for those unheard or marginalized. This passion led me to occupational therapy, a profession rooted in meaningful change. Over the years, I have witnessed individuals face profound challenges with remarkable determination and seen the transformative power of occupational therapy in restoring participation, reshaping patient journeys, and unlocking possibilities once thought unattainable. Collaborating with scholars within and beyond occupational therapy has broadened my perspectives and challenged my assumptions. Yet, it is working alongside other occupational therapists that has brought me closer to the heart of the profession. These dedicated professionals navigate complex systems with skill, compassion, and an unwavering commitment to person-centred care. Their relentless curiosity and passion continually inspire me, reaffirming why our work matters.

My journey has led me to the core of our professional knowledge: what we know, how we come to know it, and how this knowledge evolves in our work with individuals, families, and communities. As a researcher, I have devoted my career to understanding the dynamic interplay of evidence, clinician expertise, and lived experience, especially as healthcare systems face increasing pressures. This has reinforced my conviction in striking a balance between evidence-based practice and context-sensitive, compassionate care.

As occupational therapists, we find ourselves at a crucial moment. Aging populations, globalization, resource disparities, environmental issues, regulatory and workforce challenges, misinformation, the rise of artificial intelligence, and the ongoing effects of the pandemic—all in a world that urgently needs more empathy and humanity. These forces are transforming our profession, pushing us to rethink how we learn, adapt, and evolve. Yet, amid these challenges lie unprecedented opportunities. How can we sustain and advance the knowledge that defines us? How do we integrate evidence-based practice with patient voices, professional wisdom, and interdisciplinary perspectives? These questions are at the heart of this lectureship.

In the following pages, I invite us to reflect on our profession's epistemic identity—how we develop, acquire, validate, and apply knowledge; what we consider credible and reliable; and how we engage with diverse forms of knowledge both within and beyond our discipline as we support clients and prepare the next generation of occupational therapists. Our journey begins with The Evolution of Occupational Therapy Knowledge and Practice, where I will examine how knowledge in our field has evolved and the tensions that have emerged between different forms of knowledge. I will then turn to Occupational Therapy's Epistemic Identity and Its Role in Epistemic Communities, where I propose that occupational therapy is itself an epistemic community—and a vital contributor to broader interdisciplinary networks. These are groups of experts with recognized authority who collaborate to advance knowledge in their field. I will explore how our participation in such communities can enhance our engagement with multiple knowledge forms, foster innovation, improve access to rehabilitation, and shape inclusive policies that promote participation and wellbeing. In the final section, Calls to Action for Strengthening Occupational Therapists’ Role and Impact in Epistemic Communities, I will outline concrete actions to reinforce our knowledge foundations, amplify our collective voice, and expand our influence within healthcare systems and society.

## Section 1: The Evolution of Occupational Therapy Knowledge and Practice

Let us begin by walking through these two scenarios together that reflect how our knowledge has evolved over the last century.

*Imagine an early 1900s* occupational therapist assisting a patient in weaving a basket as part of their recovery. The therapist observes intently as the patient maneuvers the reeds, noting the frustration or ease on their face and how the repetitive motions calm their restless hands and anxious mind. There is no checklist or standardized measure of progress, only a deep, embodied understanding of healing through doing. Was this practice guided by intuition, by the subtle art of reading bodies and emotions? Was it shaped by emerging mental and physical rehabilitation theories, still in their infancy? Or was it led by the patient's lived experience, history, preferences, and self-perceived capacity for recovery? Perhaps it was all three, woven together as seamlessly as the fibres of the basket itself. A tapestry of multiple threads, each essential, each reinforcing the other. *Fast forward to 2025,* an occupational therapist sits across from a young single mother with rheumatoid arthritis in a quiet corner of a community health clinic. Her toddler stacks blocks nearby as she grips an ergonomic pen designed to reduce joint strain. She flexes her fingers, winces slightly, then adjusts her grip with a nod. The therapist observes not only the movement but also the hesitation, frustration, and quiet moments that reveal a deeper story of pain, adaptation, and resilience. Living in a remote community, she sees an occupational therapist infrequently. Each session must efficiently address pressing needs while anticipating future challenges. They focus on joint protection, symptom tracking to help identify flare patterns, and hands-on trial-and-error to find practical solutions. Together, they explore adaptive baby gear, fatigue management strategies, and ways to navigate parenting alone without a support network. The therapist is mindful that there is no backup—no one else to carry the groceries, lift the stroller, or handle nighttime wakeups when flare-ups make movement unbearable. However, beyond techniques and strategies, the therapist relies on something immeasurable: listening, recognizing the patient's evolving relationship with her condition, and creating a space where independence and self-efficacy take precedence over rigid protocols. Is this practice shaped by biomechanics? By emerging rehabilitation strategies? By the mother's lived experience—what works, what doesn’t, what she wishes existed? Perhaps all three are woven into a dynamic framework—a tapestry of evidence, expertise, and human connection, each reinforcing the other.

### The Evolution of Occupational Therapy's Epistemic Identity

These two scenarios, set over a century apart, illustrate the core of occupational therapy's epistemic identity: how we generate, integrate, and apply knowledge in practice (Alcoff, 2010; Fricker, 2007). They highlight how occupational therapy has always been shaped by a dynamic interplay of different knowledge sources—scientific evidence, professional expertise, and the lived experiences of the individuals we serve.

Epistemic identity is shaped by disciplinary training, cultural influences, personal experiences, and institutional norms (Alcoff, 2010). For instance, a clinician with a strong evidence-based practice epistemic identity may focus on randomized controlled trials and systematic reviews. In contrast, another clinician with a pragmatic or humanistic epistemic identity might incorporate, or even prefer, patient narratives and experiential knowledge alongside scientific data. Our epistemic identity guides how we define legitimate knowledge, assess the relevance of information to the clinical issue, negotiate expertise, and interact with other knowledge systems. This is especially important in interdisciplinary work, where different epistemic identities need to be navigated and harmonized to promote meaningful collaboration and innovation (Horn et al., 2023; Lyall et al., 2011).

### Epistemic Identity in the 1900s

In the 1900s, the occupational therapist relied heavily on embodied knowledge, intuition, and relational understanding (Andersen & Reed, 2024). Lacking standardized measures or clinical trials for guidance, they navigated patient care by interpreting bodies, emotions, and responses to activity. From its inception, the profession did not fit neatly within the biomedical model; it blended insights from nursing, orthopedics, physiotherapy, psychiatry, and social work (Dillingham, 2002; Dunton, 1919; Eldar & Jelić, 2003). This interdisciplinary foundation shaped a holistic approach, positioning purposeful activity as a core driver of health and wellbeing. The therapist's epistemic identity was grounded in practical wisdom—a deep, experience-based understanding of healing through doing. Even in its infancy, the profession had to balance what was observable, what was felt, and what was theorized.

Over time, particularly in response to the impact of war, our profession evolved (Dillingham, 2002; Eldar & Jelić, 2003). After the two World Wars, it became closely linked to the rehabilitation of injured soldiers, using activities of daily living to restore both physical function and psychological resilience (Eldar & Jelić, 2003). Early rehabilitation theories and empirical observations shaped practice, merging tacit knowledge with emerging scientific frameworks. By the 1940s, research and scientific rigor increasingly supported occupational therapy knowledge (Singal, 1987). Advances in neuroplasticity and motor learning broadened the field, while a growing emphasis on research sought to align occupational therapy with medical and scientific paradigms (Stockmeyer, 1967). By the 1970s, research organizations and professional journals reinforced positivist approaches to knowledge, prioritizing objective, measurable outcomes over tacit, intuitive, or relational forms of understanding (Mosey, 1970; Mosey, 1985).

### Epistemic Identity in 2025

In 2025, occupational therapy is well established within the health and social service sectors, showcasing a rich history that reflects an ongoing tension between scientific rigor and the art of practice. The profession has expanded in scope, technology, and evidence-based frameworks; however, fundamental epistemic tensions persist. While evidence-based practice has bolstered clinical credibility, some scholars caution that an overemphasis on measurable outcomes may overlook crucial humanistic elements, such as empathy, intuition, the art of healing, and the importance of context and clinical experience (Hinojosa, 2013; Reid et al., 2024; Whiteford, 2005). Although harder to quantify, these qualities remain vital to effective therapeutic practice.

The young mother in a remote community does not merely require research-backed interventions; she needs solutions that accommodate the messiness of real life. The therapist must integrate intuition, creativity, and clinical expertise with scientific evidence to deliver effective and meaningful care. Scientific-empirical knowledge informs her clinical decisions; joint protection strategies, rehabilitation frameworks, and symptom tracking help shape her treatment plan. Practice-based knowledge, developed through years of experience, enables her to anticipate challenges, prioritize care in a limited time, and discern when to trust her intuition over strict protocols (Higgs & Titchen, 2001; Kinsella, 2012). Patient knowledge is central; the mother's lived experience, pain management strategies, and the realities of her remote environment dictate what will be effective for her (Dumez & L’Espérance, 2024). Esthetic knowledge nurtures creativity, empathy, and emotional connection, reminding the therapist that healing encompasses not only physical, but also psychological, emotional, and spiritual dimensions (Gorodeisky & Marcus, 2022).

### Defending and Defining Our Pluralistic View of Knowledge

Occupational therapy thrives on epistemic pluralism (Zangwill, 2020), the ability to weave together these various forms of knowledge into meaningful, context-sensitive care (Kinsella, 2012). This diversity is a strength. It allows occupational therapists to address the complexities of human occupation with both adaptability and intellectual discipline. However, pluralism does not mean that all knowledge carries equal weight in every context. Without clear criteria for integration, we risk crossing a line, from thoughtful inclusion to epistemic relativism, where any perspective is treated as valid, regardless of its foundation.

We must continually grapple with critical tensions: How do we ensure that clinicians who lean on intuition still ground their decisions in rigorous evidence? When patient narratives diverge from empirical findings, how do we navigate and reconcile those perspectives? And how do we guard against misinformation or anecdote, weakening our knowledge base rather than enriching it?

For decades, occupational therapy has balanced multiple modes of knowledge, but the way we justify and apply this knowledge has evolved. Without intentional integration, we risk becoming fragmented, or worse, allowing policymakers, funders, and adjacent professions to define what constitutes legitimate knowledge in occupational therapy. The solution is not a rigid hierarchy where one form of knowledge dominates, but a structured interaction that ensures our knowledge remains inclusive, defensible, and practical. Our strength lies not in rejecting empirical knowledge, but in rigorously and intentionally bringing it together with wisdom gained from practice, relational insight, and lived experience. False dichotomies—science versus experience, measurable outcomes versus meaningful participation—hold us back. We do not have to choose; we have to integrate. The real challenge is not only to recognize the plurality of knowledge, but to intentionally mobilize it and embed it in the systems that shape health practices and policies. How, then, can we ensure that this pluralistic but rigorous approach is not only recognized but supported in research, practice, and policy? This is where epistemic communities become essential.

More than mere knowledge-sharing networks, epistemic communities define what constitutes legitimate (Haas, 1992). They validate and mobilize it, integrating diverse ways of knowing into systems of influence. By guaranteeing that knowledge remains responsive to social and policy contexts, they advocate for its incorporation into healthcare systems in rigorous and acknowledged ways.

## Section 2: Occupational Therapists’ Role in Epistemic Communities: Contributing to Policy and Practice

### Introduction to Epistemic Communities

Epistemic communities have long played a crucial role in addressing complex global challenges. For instance, collaborations among biologists, environmental economists, and policy experts in climate science have shaped significant international policies, such as the Kyoto Protocol (Nations, 1998) and the Paris Agreement (Nations, 2016). Similarly, the World Health Organization's reliance on networks of epidemiologists, public health experts, and medical professionals has been crucial in coordinating responses to pandemics such as HIV/AIDS, SARS, and COVID-19 (Mediterranean, 2024). These communities adeptly incorporate viewpoints from various fields, including basic sciences, social sciences, law, and economics, to create nuanced strategies that diminish inequalities, promote inclusive policies, and drive systemic change.

In the late twentieth century, sociologist E. Haas introduced the concept of epistemic communities *as “networks of professionals with recognized expertise in a specific domain and an authoritative claim to policy-relevant knowledge”* (Haas, 1992, p. 3).

#### Is Membership in an Epistemic Community a Matter of Expertise, Recognition, or Power?

Cross (Mai’a K, 2013) argues that epistemic communities are not simply the entirety of any profession, nor are they limited to members of a profession. While these communities are often linked with scientists, they also notably include nonscientific experts. Such groups can consist of diverse professionals, including policy analysts, advocates, and other key knowledge holders, not just those in scientific or technical fields. Importantly, epistemic communities are legitimate because their members are seen as credible in a particular area and can reach a consensus on essential issues. However, their influence is not inherent; it is shaped by evolving power dynamics and political factors that determine how their contributions are recognized and implemented in policymaking (Haas, 1992).

### Key Criteria of an Epistemic Community

For epistemic communities to work effectively, they must meet four key criteria—expressed through seven modes of operation—that enable policy influence and meaningful collaboration toward common goals (Haas, 1992; Smirnova & Yachin, 2015).

1. *A shared set of normative and principled beliefs or shared values:* A key trait of an epistemic community is its commitment to shared values and guiding beliefs. N*ormative beliefs* unite members around shared goals and ethical principles, such as occupational therapists’ commitment to patient-centred and equitable rehabilitation. *Principled beliefs* shape how information is understood and applied, such as the assumption that evidence-based practice improves patient outcomes. The community operates on two central tenets: *a shared commitment to truth,* recognizing that truth can be understood in different ways; prioritizing credible scientific knowledge over personal or political interests; and a*dherence to shared values*, ensuring a consistent, value-driven approach to problem-solving and policy advocacy.
2. *Shared causal beliefs or shared understanding:* The second characteristic is the community's shared understanding of the root causes behind the challenges it seeks to address. This enables members to create targeted interventions and policy solutions and is operationalized in two ways: *Interdisciplinary Collaboration,* where experts from occupational therapy, public health, law, and education collaborate on improving access to services that optimize participation and reduce health disparities, and *Political Influence,* where a collective commitment to an issue mobilizes support, shapes policy, and drives systemic change.
3. *Shared notions of validity:* A third characteristic is a shared understanding of valid and reliable knowledge. Common standards guide members in evaluating evidence, tackling problems, and making policy recommendations. This criterion manifests in two ways: *personal responsibility—e*ach member is accountable for contributing rigorous evidence and professional expertise to inform decision-making—and *adherence to shared criteria for valid knowledge—ensuring* consensus on what qualifies as reliable knowledge—whether based on scientific research, lived experiences, or clinical best practices—maintaining consistency in how evidence is assessed and applied.
4. *A shared commitment to action—common policy enterprise:* The final trait centres on a shared mission to tackle challenges through collective expertise. Members collaborate to create a tangible impact, utilizing their knowledge to improve wellbeing and drive change. Key modes of operation include a *focus on global* issues that ensure their efforts address long-term, systemic problems affecting human development and societal wellbeing and self-o*rganization,* which allows members to work autonomously within their specialized areas while strategically collaborating to produce transformative solutions.

### Occupational Therapy AS an Epistemic Community

Occupational therapists actively generate, validate, and disseminate knowledge—processed we engage in almost daily. As such, occupational therapy functions *as* an epistemic community: an *internal* epistemic community that enhances the legitimacy and advancement of our profession. Here are three examples of how we operate as an epistemic community:

- *Research networks:* Through national and international collaborations, occupational therapists contribute to the generation of evidence using a variety of approaches. These approaches include studies that utilize randomized controlled trials as well as more participatory and inclusive research methods. Such collaborations enhance our understanding of the determinants of human occupation and allow us to develop and test interventions that improve client outcomes.
- *Professional associations:* Organizations like the Canadian Association of Occupational Therapists (Therapists, 2024) and the World Federation of Occupational Therapists (Therapists, 2025) promote knowledge sharing through conferences, position statements, and practice guidelines that influence the profession.
- *Educational frameworks*: In academic settings, occupational therapy programs prepare students to critically appraise and integrate evidence into diverse practice environments, ensuring that graduates can provide high-quality care and that the profession continues to evolve based on sound knowledge (Programs, 2025).

#### Occupational Therapy AS PART OF a Broader Interdisciplinary Epistemic Network

Occupational therapists also play a vital role in broader interdisciplinary epistemic communities. Just as climate scientists and policymakers join forces to tackle environmental and public health challenges, occupational therapists work alongside policymakers, engineers, other healthcare professionals, managers, economists, public health officials, social scientists, and citizens. Through interdisciplinary collaboration, advocacy, and knowledge mobilization, we actively shape healthcare policy, education, and practice to enhance access to rehabilitation and drive systemic change. We offer a unique epistemic contribution to these broader communities by incorporating our occupation-based perspectives, patient-centred approaches, and focus on participation as a health outcome. This represents an *external epistemic community.* Here is an example: I am part of an epistemic community dedicated to advancing person-centred, evidence-based rehabilitation care for millions of people in Canada who need it. While I had been working to bridge perspectives from education, practice, and policy to promote evidence-based practices, I recognized that more was needed. To strengthen these connections, several colleagues and I collaborated to explore how we could do this more effectively. Over time, we fostered a diverse community of researchers across health services, rehabilitation, implementation science, sociology, anthropology, political science, health economics, geography, and epidemiology. We collaborated with industry stakeholders, academic institutions, patient representatives, professional associations, regulators, rehabilitation hospitals, community organizations, policymakers, government officials, and Indigenous partners and communities. Our community also encompasses frontline professionals, including occupational therapists, physiotherapists, chiropractors, nurses, physicians, and psychologists, who work in primary care, rehabilitation, and community settings. How do we qualify as an epistemic community? *Shared Normative Beliefs:* We are united by a core commitment to person-centred, evidence-based rehabilitation care. We believe that access to timely, high-quality rehabilitation is a right, not a privilege, and that interdisciplinary collaboration is essential to achieving this. These shared values guide our efforts to bridge the gaps between research, policy, and practice.

*Shared Causal Beliefs:* We acknowledge that barriers to accessing rehabilitation extend beyond clinical factors and are intricately woven into political, economic, and social frameworks. Tackling these obstacles necessitates a multisectoral approach, incorporating insights from health services research, implementation science, sociology, and policy while actively involving Indigenous partners, patient representatives, industry leaders, and government officials. By integrating these viewpoints, we aim to develop evidence-informed, contextually relevant solutions that enhance access to rehabilitation and improve outcomes across Canada.

*Shared Notions of Validity*: We uphold rigorous, interdisciplinary knowledge production that values both qualitative and quantitative evidence, as well as contextual differences and lived experiences. We acknowledge that success is not measured solely by clinical trials, but also by real-world impact, community engagement, and culturally responsive approaches.

*Common Policy Enterprise:* We are committed to developing sustainable care models that ensure timely and equitable access to rehabilitation. Our policy-driven mission promotes the integration of rehabilitation into primary and community care, strengthens Indigenous-led health initiatives, and prioritizes rehabilitation in healthcare policy. By collaborating with policymakers, regulators, and professional associations, we aim to shape the future of rehabilitation in Canada. Today, our epistemic community comprises nearly 60 dedicated individuals who, particularly over the past 2 years, have worked tirelessly toward a common goal: ensuring that individuals receive rehabilitation services when they need them most, at the right time, and from the appropriate healthcare professionals.

### From Concept to Action: Why This Matters for Occupational Therapy's Future

Epistemic communities are not just abstract networks—they shape professional identity, influence healthcare policy, and drive tangible change in the real world. Occupational therapy stands at a crossroads: we can actively shape our role in evolving healthcare systems, or risk being defined by others. Three forces are driving this urgency: (1) profession-level; (2) knowledge-level; and (3s Systems-level.

Profession-level: The healthcare landscape is evolving, and occupational therapy must clearly define its role, or risk being defined by others. As care models shift, funding structures change, and privatization expands, our profession cannot afford to be passive. For instance, a key opportunity lies in primary care integration, where the expertise of occupational therapists in preventive and community-based care is invaluable. However, being valued is not enough; we must proactively assert our place in these models. Professions that strategically position themselves in growing areas gain influence in funding, policymaking, and system design. Without clear advocacy, our profession risks being marginalized or absorbed into broader disciplines, which would undermine its distinct contributions. At the same time, our internal debates, such as the discussion on generalists versus specialists (Foto, 1996), should not divide us. Instead of viewing this as a liability, we ought to leverage it to clarify and strengthen our position in healthcare. Health policy favors organized, strategic professions over a fragmented approach.

Knowledge-level: Interdisciplinary knowledge sharing is vital in occupational therapy; however, professional silos, regulatory constraints, and rigid definitions of evidence frequently hinder collaboration. Take, for example, the COVID-19 pandemic, which necessitated rapid innovation, accelerating the adoption of telehealth and requiring cross-disciplinary collaboration to maintain care (Omboni et al., 2022; Sharma et al., 2022; Shaver, 2022). Initially, occupational therapists faced obstacles—diverse professional languages, regulatory frameworks, and funding models—especially in stroke rehabilitation, where coordination was crucial (Harahsheh et al., 2022; Hubert et al., 2021; Kimmel et al., 2024). Nevertheless, these challenges also fostered innovation, leading to more integrated systems, updated telehealth guidelines, and enhanced collaboration. Occupational therapists played a pivotal role in adapting service delivery, strengthening networks, and incorporating rehabilitation into virtual care models. Despite these contributions, insights from occupational therapy were often absent from policy decisions, revealing a deeper issue: What constitutes valid knowledge?

Traditional evidence-based practice prioritizes clinical trials, ensures rigor, and minimizes bias (Library, 2025). However, lived experience, qualitative research, and participatory approaches provide equally vital insights (Baum et al., 2006; Cargo & Mercer, 2008; Greenhalgh & Papoutsi, 2018). The pandemic highlighted the limitations of narrow evidence-based practice frameworks; while the effectiveness of telehealth was confirmed, evaluations rarely considered accessibility, long-term rehabilitation needs, or culturally responsive care. If occupational therapy aims to influence healthcare transformation, we must challenge outdated definitions of evidence, advocate for more inclusive knowledge systems, and ensure that our expertise informs—not merely responds to—system-wide change.

Systems-level: Healthcare is evolving—aging populations, globalization, workforce shortages, and resource constraints are reshaping service delivery (Elmslie, 2012; Kolb, 2018; Wong, 2014). COVID-19 accelerated these shifts, highlighting both the system's vulnerabilities and the opportunities for occupational therapy to lead innovation, strengthen patient-centred care, and influence policy (Nana-Sinkam et al., 2021). As life expectancy increases, so does the demand for sustainable rehabilitation models (Lindgren, 2016; Meinow et al., 2022; Organization, 2017). Artificial intelligence (AI) is transforming healthcare, but occupational therapist must ensure that technology enhances—not replaces—human-centred care, safeguarding therapeutic relationships, and clinical expertise.

Occupational therapy thrives at the intersection of clinical care, education, policy, and research. However, structural silos can hinder the implementation of best practices (Amri et al., 2022). Stroke rehabilitation illustrates this issue—while evidence supports comprehensive, patient-centred recovery models, policy barriers often hinder their real-world adoption (Baatiema et al., 2017; Cormican et al., 2023). Occupational therapists need to engage with hospital administrators, policymakers, and funders to position rehabilitation as an investment, rather than an afterthought. By bridging gaps across healthcare, education, and social services, occupational therapy can drive system-wide change and reinforce its role in shaping the future of healthcare.

### Moving Forward: Strength in Integration

Our strength lies in integrating various forms of knowledge—science, practice, lived experience, and community wisdom. However, occupational therapy encompasses more than just knowledge; it focuses on connection—bridging disciplines, translating insights into action, and promoting participation. These strengths empower us to tackle global challenges. We are both a distinct professional community and an essential part of broader interdisciplinary networks. This dual role is our strength—but only if we embrace it. Clarifying this dual role enables us to define our identity instead of allowing others to do so. Rather than questioning our place, we must assert our leadership in shaping health, wellbeing, and justice.

## Section 3: Calls to Action: Strengthening Occupational Therapists’ Role and Impact in Epistemic Communities

Occupational therapy is at a crucial juncture—not as a challenge, but as an opportunity to lead. The core values that healthcare now promotes—personalized care, holistic recognition of individuals, relationship-centred practice, and high-quality evidence—have long been embedded in our profession. To keep occupational therapy and rehabilitation at the forefront of healthcare, we must move from recognition to action: sharing our expertise, embracing diverse ways of knowing, advocating for transformative policies, and ensuring our voices shape broader healthcare conversations. We already belong in these spaces. Now is the time to step forward with purpose and lead.

Next, I suggest three key calls to action to strengthen our knowledge base, amplify our collective voice, and expand our influence in shaping the future of the profession, rehabilitation and healthcare more broadly.

### Call #1: Strengthening Our Knowledge Base: Epistemic Humility, Expanding Evidence, Bridging Research-Practice Gaps

#### Epistemic Humility

At the heart of an epistemic community is a commitment to mutual respect, collaboration, and epistemic humility—the recognition that knowledge is not solely based on individual expertise but is shaped by collective wisdom (Kidd, 2017; Maduro, 2012). It involves valuing voices traditionally excluded from the planning and provision of care and integrating insights from public health, urban planning, economics, integrative medicine, engineering, sociology, and geography, among others. These partnerships challenge our assumptions and broaden our understanding of important phenomena. How can we cultivate this humility? I suggest a six-step, action-oriented framework for integrating diverse knowledge into occupational therapy education, practice, and policy. This process must begin early and remain central to our profession, necessitating structured engagement in classrooms, fieldwork, and professional discussions among educators, clinicians, managers, and clients:

- Listen (hear): Authentic listening requires openness, patience, and a willingness to engage with perspectives that challenge our own.
- Tolerate (hold space): Meaningful dialogue means making space for disagreement without forcing consensus.
- Reflect (pause and consider): Critically assess how knowledge is constructed, who is included, and what assumptions shape understanding.
- Learn (grow): Knowledge is fluid and evolving, not fixed or certain. Growth comes from embracing this reality.
- Attempt (try with courage): Change requires action. Take risks, experiment with new models, and view failure as an integral part of the learning process.
- Assess (recalibrate). Evaluation is about learning, not just measuring success. Understand what works, what does not, and why.

#### Expanding Evidence and Bridging Research-Practice Gaps

Occupational therapists play a vital role in creating, sharing, and applying knowledge in all its forms. We must engage at every step, ensuring that diverse knowledge systems are acknowledged and that new insights reflect patients’ lived experiences. How can we achieve this as part of our epistemic communities?

1. *Knowledge creation*: We must produce knowledge that captures the complexities of (dis)ability, function, and participation through participatory, practice-based, and co-creation methods. This necessitates methodological innovation, community-led research, and real-world evidence to ensure relevance and inclusivity. Knowledge creation must embrace non-Western frameworks and incorporate diverse epistemic perspectives into rehabilitation research. This entails engaging Indigenous health leaders, co-developing community-driven approaches, and drawing insights from global health systems.
2. Knowledge dissemination: Knowledge must reach the right audiences in ways that promote action. We need to shape not just who receives it, but also how it is communicated and applied. Effective dissemination ensures that evidence extends beyond academic realms to impact practice and policy.
3. Knowledge implementation: Implementation is not optional—it is central to our role. We must actively support the uptake of the knowledge we generate, ensuring it translates into meaningful change in practice and policy.

Two critical issues influence all three stages: (1) *Recognizing power structures*—Bridging the research-practice gap necessitates addressing who controls knowledge and whose expertise is valued (Kitto et al., 2012; Rycroft-Malone et al., 2016). We must challenge these hierarchies to create inclusive, patient-centred knowledge systems; and (2) *Promoting critical engagement and reflexivity*—Knowledge evolves through critical reflection, dialogue, and adaptation (Ng et al., 2022; Sherwood, 2024). Incorporating reflexivity into education, research, and practice allows us to critically examine our assumptions, values, and positionality in care. This enhances our ability to challenge biases, respond to emerging needs, and uphold ethical, socially just, and client-centred rehabilitation. *A word of caution:* Broadening our understanding of knowledge does not mean abandoning evidence-based practice. While it can sometimes marginalize experiential or tacit knowledge, it remains a critical safeguard against bias and misinformation. We must expand our definition of evidence while recognizing the limitations of traditional empirical approaches. If evidence continues to serve as the standard for legitimacy, we must articulate this reality, acknowledge its constraints, and move forward unapologetically—not to replace it, but to reconcile.

### Call #2: Strengthening Our Collective Voice: Clarifying Our Identity, Reducing Fragmentation, Shaping Public Perception

#### Clarifying Our Identity, Reducing Fragmentation, and Shaping Public Perception

If we are fractured internally, we undermine our epistemic community and our capacity to advocate effectively in interdisciplinary ones. Our adaptability is a strength, but our collective influence diminishes without clarity regarding our roles, scope, and contributions. To amplify our impact, we must unite around a shared vision—one that integrates research, practice, and the social justice principles that underpin our work. Unity, however, does not require rigid standardization; instead, it entails shared principles, core competencies, and an appreciation of diverse contexts. By co-creating guiding principles that define our impact, expertise, and interventions, we enhance our influence on policy, funding, and innovation. This strategic initiative enables us to collaborate across disciplines and expand our impact on mental health, dementia care, rehabilitation technologies, and community-based disability inclusion. Here is where we must concentrate our efforts:

*1. Leverage differences for innovation*: Regional disparities, role confusion, and siloed practice areas weaken our influence. However, these differences can also be strategic advantages. Rather than avoiding difficult conversations, we must analyze the root causes of fragmentation, facilitate structured debates to bridge gaps, and turn diverse perspectives into innovative solutions for practice, policy, and education. Cohesion is not uniformity—it is about strategically aligning our expertise to advance the profession.
*2. Establish ongoing spaces for dialogue*: Change does not occur through a single conversation. To influence policy and strategy, we require structured, sustained forums. This involves creating dedicated discussion spaces within professional associations, interdisciplinary forums, and the national policy arena, fostering interdisciplinary collaboration, and ensuring occupational therapists actively participate in national policy discussions. If we are not included in the conversation, we will be omitted from the decisions shaping the future of healthcare.
*3. Clarify our unique value, strengthen our advocacy:* What distinguishes occupational therapy from other professions? To enhance our influence, we must clearly define and communicate our core competencies, align our advocacy with key healthcare priorities, and ensure our message is consistent, persuasive, and data driven. By refining how we convey our value, we reinforce our professional standing while maintaining our distinctiveness.

### Call #3: Strengthening Our Influence: Collaboration, Policy Impact and Leadership

Occupational therapists have the expertise to influence health policy but remain underrecognized. Without targeted advocacy, the profession risks being left out of important healthcare decisions. Our role goes beyond clinical work—we help set priorities, develop interventions, and manage resources. System change requires occupational therapy at the decision-making table, not on the sidelines. This shift must start early, embedding policy engagement and advocacy skills into occupational therapy education from the beginning.

#### Collaboration as a Catalyst for Influence

Collaboration in healthcare is not optional; it is essential. Occupational therapy, by its very nature, is inherently collaborative, requiring engagement with patients, physicians, nursing home staff, and other professionals. It operates at the intersection of care, bridging disciplines and ensuring continuity across treatment phases. Occupational therapists must move beyond good intentions by actively defining and communicating their unique contributions while fostering sustained partnerships that create measurable impacts locally, nationally, and internationally. Strengthening these collaborations lays the foundation for meaningful policy change. Here are examples of how we can put it into practice.

*Interdisciplinary engagement for system integration*: To secure a role in shaping healthcare, we must engage across various disciplines and sectors. This entails:

- Co-hosting workshops with hospital administrators and rehabilitation teams to enhance stroke care.
- Participating in healthcare innovation roundtables to highlight the impact of occupational therapy on reducing hospital readmissions.
- Establishing standing committees with primary care providers to improve early referrals for musculoskeletal conditions.

*Institutional partnerships for sustainable impact*: Achieving sustainable influence necessitates structural collaboration with key organizations.

- Collaboratively developing a national framework for rehabilitation service delivery with the Canadian Association of Occupational Therapists.
- Integrating occupational therapy content into interdisciplinary university health programs to ensure future clinicians appreciate its value.
- Partnering with patient advocacy groups, such as the Patient Advisory Network, to co-create resources focused on rehabilitation access.

*Global networks for broader reach:* Occupational therapy extends beyond national boundaries. Examples include:

- Partnering with the WHO on healthy aging initiatives.
- Contributing to World Federation of Occupational Therapists (WFOT) rehabilitation guidelines.
- Engaging in global research consortia, such as the Global Alliance for Rehabilitation and Assistive Technology (Headquarters, 2019).

While collaboration is essential for enhancing our impact, influencing policy necessitates a strategic shift in how we integrate rehabilitation within healthcare systems.

### From Influence to Leadership: Advancing Policy Impact

Occupational therapists need to move from the margins to the forefront by reimagining rehabilitation as a key health priority and addressing the acute care bias that often sidelines it. We must establish rehabilitation as essential to the sustainability of the health system and advocate for its integration into policies.

Embedding occupational therapy in policy agendas: Occupational therapists do not need to be policy experts to effect change. By engaging with key frameworks and funding decisions, we can ensure that rehabilitation is recognized and adequately resourced. This means: Advocating for funding allocations that reflect rehabilitation's role in prevention and long-term care, participating in national and global policy discussions, such as WHO's *Rehabilitation 2030* (Organization, 2025), to embed occupational therapy in health reform but also, leveraging economic and policy research to demonstrate the cost-effectiveness of occupational therapy in primary care, mental health, and aging.

Expanding the definition of evidence in policymaking: Policy decisions often prioritize clinical trials and biomedical metrics; however, rehabilitation must also integrate patient narratives and lived experiences, as well as community-driven interventions that capture real-world impact, and health system research demonstrating the practical benefits of rehabilitation.

Bridging research and policy for real-time impact: Closing the Gap between Research and Practice Requires Robust Knowledge-Sharing Mechanisms. Occupational therapists must help translate rehabilitation research into actionable policy recommendations, engage policymakers directly to ensure evidence informs decision making and establish cross-sector networks to position occupational therapy as central to healthcare transformation. By combining strategic collaboration with active policy engagement, occupational therapists can shift from being participants to shaping the future of rehabilitation policy.

## Conclusion

### Embodying the Knowledge We Hold: From Identity to Action

Occupational therapy has continually evolved to meet society's needs—from moral treatment to wartime rehabilitation, evidence-based practice, and advanced technology. What started as a radical concept—that meaningful activity fosters healing—has transformed into a discipline grounded in science, clinical wisdom, and personal experience. As healthcare becomes increasingly complex, we do not require absolute certainty, but we do need clarity. We are not merely service providers—we are knowledge stewards, policy influencers, and advocates for occupational justice. Yet, as we progress, we must ask ourselves: Are we evolving intentionally or simply reacting? Occupational therapy is more than just a profession; it forms an epistemic community unified by shared expertise and a dedication to understanding how occupation affects wellbeing. However, our collective beliefs alone are insufficient. The challenges ahead—health inequities, evolving care models, and AI-driven decision making—demand a coherent, confident, and adaptable profession. If we fail to assert our space, others will define it for us. Our role should not be limited to technical tasks but rather should be connected to the humanistic and contextual knowledge that enhances our effectiveness.

Why should we be optimistic about the power of our knowledge community? Because I have seen us in action.

*In education:* We are interrogating curricula, exposing the hidden curriculum, and evolving teaching and assessment methods. We are bridging academia and practice through deeper engagement with students, clinical sites, and preceptors.

*In practice:* We are partnering with Indigenous communities, nongovernmental organizations (NGOs) and community organizations to co-create culturally responsive care. We are leading aging, child, and mental health initiatives, ensuring participation on people's terms. We are making an impact—not only addressing impairment-level outcomes but also fostering occupational engagement and participation.

*In policy:* We are engaging policymakers and working with the UN, WFOT, and WHO. Where we once knocked on policymakers’ doors, they now knock on ours.

I leave you with these questions—not as abstract reflections, but as imperatives: How can we better support the next generation of occupational therapists in developing critical, reflective, and adaptive knowledge and skills? How can we strengthen our collective impact by enhancing our involvement in interdisciplinary collaboration in healthcare, education, and policy? How can we take a more proactive approach to shaping policy discussions surrounding rehabilitation, participation, and health equity, leveraging significant moments in society? What commitments will we make—both individually and collectively—to embody the values of an epistemic community in action?

*Moving forward with confidence:* Our profession thrives on striking a balance between structure and flexibility, tradition and innovation, as well as science and art. This is not a weakness; it represents our greatest strength. It keeps us adaptable while grounding us in core principles. We value pluralism, humility, collaboration, reflexivity, and the courage to engage in constructive disagreement. We must move forward with confidence, vision, and the collective strength of our profession. The knowledge we hold is powerful—but only if we use it together. By embracing our full epistemic identity, we strengthen our profession, enhance participation, improve quality of life, and advance justice and equity across individuals, communities, and entire systems.
