# Supplementary material for: The Knowledge We Hold: Exploring Occupational Therapy's Epistemic Identity and the Balance Between Evidence and Practice
Source: Can J Occup Ther. 2025 Oct 29;92(4):258–81. doi: 10.1177/00084174251383259 (PMC12799800; doi:10.1177/00084174251383259)
Supplement: Related texts

# Research article: Les savoirs que nous portons : explorer l’identité épistémique de l’ergothérapie et l’équilibre entre les données probantes et la pratique

**Authors:** Aliki Thomas

**Keywords:** Communautés épistémiques, humilité épistémique, influence sur les politiques, pluralisme, transformation du système.

## Abstract

Depuis fort longtemps, l’ergothérapie puise sa force dans l’intégration de multiples sources de savoirs : données probantes, expertise clinique, récits de clientèle et sagesse incarnée. Cette conférence interroge l’identité épistémique de la profession : comment nous développons, validons et mobilisons les connaissances pour relever des défis de santé et sociaux complexes. À partir d’exemples historiques et contemporains, l’autrice examine l’évolution des savoirs, retraçant le passage des soins intuitifs et relationnels vers des modèles fondés sur des données probantes, jusqu’au paysage épistémique pluraliste actuel. Elle soutient que l’ergothérapie agit comme une communauté épistémique, détentrice d’un savoir légitime et politiquement pertinent, active au sein des réseaux interdisciplinaires, capable d’orienter les modèles de soins, les réformes politiques et les transformations des systèmes. Son influence repose toutefois sur l’humilité épistémique, la collaboration stratégique et l’engagement à articuler recherche, pratique professionnelle et vécu expérientiel. L’autrice propose trois appels à l’action : (1) enrichir notre base de savoirs par le pluralisme et la réflexivité; (2) amplifier notre voix collective en clarifiant notre identité professionnelle et en réduisant la fragmentation; et (3) accroître notre influence politique grâce à une collaboration et un leadership ciblés. Dans un système de santé en pleine évolution, l’ergothérapie doit affirmer son rôle non seulement comme prestataire de services, mais également comme gardienne d’un savoir intégré, ancré dans les contextes, et orienté vers la participation, l’équité et la justice.

## Introduction

Dès mon plus jeune âge, j’ai ressenti un profond appel à aider les autres – à lutter contre l’injustice et à défendre les personnes marginalisées ou celles que l’on n’entend pas. Cette passion m’a menée jusqu’à l’ergothérapie, une profession enracinée dans le changement significatif. Au fil des ans, j’ai vu des individus faire face à de profonds défis avec une détermination remarquable, et j’ai constaté le pouvoir transformateur de l’ergothérapie pour restaurer la participation, remodeler les parcours des personnes et libérer un potentiel autrefois considéré comme inaccessible. La collaboration avec le milieu de la recherche au sein et au-delà de l’ergothérapie a élargi mes perspectives et remis en question mes présupposés. Toutefois, c’est de travailler aux côtés d’autres ergothérapeutes qui m’a rapprochée du cœur de la profession. Ces professionnelles et professionnels dévoués naviguent dans des systèmes complexes avec compétence, compassion, et un engagement inébranlable à l’égard des soins axés sur la personne. Leur curiosité et leur passion m’inspirent continuellement, réaffirmant l’importance de notre travail.

Mon parcours m’a menée jusqu’au cœur de notre savoir professionnel : ce que nous savons, comment nous en arrivons à le savoir, et comment ce savoir évolue dans notre travail avec les individus, les familles et les communautés. En tant que chercheuse, j’ai consacré ma carrière à comprendre l’interaction dynamique du savoir, des données probantes, de l’expertise clinique et de l’expérience vécue, en particulier dans un contexte où les systèmes de soins de santé sont confrontés à des pressions croissantes. Cela m’a confirmé l’importance d’un équilibre entre la pratique fondée sur les données probantes et les soins compatissants et adaptés au contexte.

En tant qu’ergothérapeutes, nous nous trouvons à un moment crucial. Le vieillissement des populations, la mondialisation, les disparités en matière de ressources, les enjeux environnementaux, les défis réglementaires et de main-d’œuvre, la désinformation, la montée de l’intelligence artificielle et les effets persistants de la pandémie – le tout dans un monde qui a urgemment besoin de plus d’empathie et d’humanité – sont des forces qui transforment notre profession et nous poussent à repenser notre façon d’apprendre, de nous adapter et d’évoluer. Pourtant, au milieu de ces défis, il y a des occasions sans précédent à saisir. Comment pouvons-nous entretenir et faire avancer le savoir qui nous définit? Comment intégrer la pratique fondée sur les données probantes aux voix des personnes patientes, à la sagesse professionnelle et aux perspectives interdisciplinaires? Ces questions sont au cœur de la présente conférence.

Dans les pages qui suivent, je nous invite à réfléchir à l’identité épistémique de notre profession – comment nous développons, acquérons, validons et appliquons le savoir; ce que nous considérons comme crédible et fiable; et comment nous nous engageons avec diverses formes de connaissances à l’intérieur et à l’extérieur de notre discipline alors que nous soutenons la clientèle et préparons la prochaine génération d’ergothérapeutes. Nous commencerons par aborder *l’évolution du savoir et de la pratique en ergothérapie*, alors que j’examinerai la manière dont les connaissances dans notre domaine ont évolué, et les tensions qui ont émergé entre les différents modes de connaissance. Je me pencherai ensuite sur *l’identité épistémique de l’ergothérapie et son rôle dans les communautés épistémiques*. Je soutiendrai que l’ergothérapie est elle-même une communauté épistémique, et apporte une contribution essentielle à des réseaux interdisciplinaires plus larges. Ces communautés et réseaux sont des groupes d’expertes et d’experts dotés d’une autorité reconnue qui collaborent pour faire avancer les connaissances dans leur domaine. J’explorerai comment notre participation à de telles communautés peut améliorer notre engagement avec de multiples formes de connaissances, favoriser l’innovation, améliorer l’accès à la réadaptation et façonner des politiques inclusives qui favorisent la participation et le bien-être. Dans la dernière section, *Appels à l’action pour renforcer le rôle et l’impact des ergothérapeutes dans les communautés épistémiques*, je présenterai des actions concrètes pour renforcer les bases de notre savoir, amplifier notre voix collective et étendre notre influence au sein des systèmes de soins de santé et de la société.

## Section 1 : L’évolution du savoir et de la pratique en ergothérapie

Commençons par parcourir ensemble deux scénarios qui reflètent l’évolution de notre savoir au cours du dernier siècle.

Imaginez une ergothérapeute du début des années 1900 en train d’aider une patiente à tisser un panier dans le cadre de son processus de rétablissement. L’ergothérapeute observe attentivement la patiente pendant qu’elle manipule les lanières, notant la frustration ou l’aisance sur son visage et la manière dont les mouvements répétitifs calment ses mains agitées et son esprit anxieux. Il n’y a pas de liste de contrôle ou d’évaluation standardisée des progrès accomplis, seulement une compréhension profonde et incarnée de la guérison par la réalisation d’activités. Cette pratique est-elle guidée par l’intuition, par l’art subtil d’interpréter les corps et les émotions? A-t-elle été façonnée par les théories émergentes de la réadaptation physique et mentale, encore à leurs balbutiements? Est-elle plutôt guidée par l’expérience vécue de la patiente, ses antécédents, ses préférences et sa perception de sa capacité à se rétablir? Peut-être que tous ces aspects sont présents, tissés ensemble aussi harmonieusement que les fibres du panier lui-même, en une tapisserie faite de multiples fils, chacun essentiel, chacun renforçant l’autre.

De retour en 2025. Une ergothérapeute est assise en face d’une jeune mère célibataire vivant avec la polyarthrite rhumatoïde, dans un coin tranquille d’une clinique de santé communautaire. Son jeune enfant empile des blocs à proximité, tandis qu’elle saisit un stylo ergonomique conçu pour réduire les douleurs articulaires. Elle fléchit les doigts, grimace légèrement, puis ajuste sa prise avec un hochement de tête. L’ergothérapeute observe non seulement le mouvement, mais aussi l’hésitation, la frustration, et les moments calmes qui révèlent une histoire plus profonde de douleur, d’adaptation et de résilience. Vivant dans une communauté éloignée, la patiente n’a pas souvent l’occasion de voir l’ergothérapeute. Chaque séance doit répondre efficacement aux besoins pressants, tout en anticipant les défis futurs. Les séances se concentrent sur la protection des articulations, le suivi des symptômes pour aider à identifier les tendances des poussées, et des essais et erreurs dans le but de trouver des solutions pratiques. Ensemble, la patiente et l’ergothérapeute explorent des équipements adaptés pour prendre soin du bébé, ainsi que des stratégies de gestion de la fatigue et de la parentalité en l’absence d’un réseau de soutien. L’ergothérapeute sait bien qu’il n’y a pas d’option de rechange – personne d’autre n’est là pour porter l’épicerie, soulever la poussette, ou gérer les réveils nocturnes lorsque les poussées rendent les mouvements insupportables. Cependant, au-delà des techniques et des stratégies, l’ergothérapeute s’appuie sur quelque chose d’impossible à mesurer : l’écoute, la reconnaissance de la relation évolutive de la patiente avec sa maladie, et la création d’un espace où l’indépendance et l’auto-efficacité priment sur les protocoles rigides. Cette pratique est-elle façonnée par la biomécanique? Par des stratégies de réadaptation émergentes? Par l’expérience vécue de la mère – qu’est-ce qui fonctionne, qu’est-ce qui ne fonctionne pas, qu’est-ce qu’elle aimerait qu’il existe? Peut-être que ces trois aspects sont entrelacés dans un cadre dynamique – une tapisserie de données probantes, d’expertise et de lien humain, chacun renforçant l’autre.

### L’évolution de l’identité épistémique de l’ergothérapie

Ces deux scénarios, séparés par plus d’un siècle, illustrent ce qui constitue le cœur de l’identité épistémique de l’ergothérapie : comment nous générons, intégrons et appliquons des connaissances dans la pratique (Alcoff, 2010; Fricker, 2007). Ils soulignent comment l’ergothérapie a toujours été façonnée par l’interaction dynamique de différentes sources de connaissances – preuves scientifiques, expertise professionnelle et expériences vécues des personnes que nous servons.

L’identité épistémique est façonnée par la formation disciplinaire, les influences culturelles, les expériences personnelles et les normes institutionnelles (Alcoff, 2010). Par exemple, un ergothérapeute dont l’identité épistémique est fortement ancrée dans la pratique fondée sur les données probantes peut se concentrer sur les essais contrôlés avec groupe témoin aléatoire et les revues systématiques. En revanche, un autre ergothérapeute, avec une identité épistémique pragmatique ou humaniste, pourrait incorporer, ou même préférer incorporer, les récits des personnes patientes et le savoir expérientiel aux côtés des données scientifiques. Notre identité épistémique guide la façon dont nous définissons les connaissances légitimes, évaluons la pertinence de l’information par rapport à la question clinique, négocions l’expertise et interagissons avec d’autres systèmes de connaissances. Cela est particulièrement important dans le travail interdisciplinaire, où différentes identités épistémiques doivent cohabiter et être harmonisées pour promouvoir une collaboration et une innovation significatives (Horn et al., 2023; Lyall et al., 2011).

### L’identité épistémique dans les années 1900

Dans les années 1900, les ergothérapeutes s’appuyaient fortement sur les connaissances incarnées, l’intuition et la compréhension relationnelle (Andersen & Reed, 2024). Faute d’évaluations standardisées ou d’essais cliniques pour s’orienter, les ergothérapeutes s’efforçaient d’interpréter les corps, les émotions et les réponses à l’activité. Dès sa création, la profession ne s’inscrivait pas parfaitement dans le modèle biomédical; elle combinait des connaissances issues des soins infirmiers, de l’orthopédie, de la physiothérapie, de la psychiatrie et du travail social (Dillingham, 2002; Dunton, 1919; Eldar & Jelić, 2003). Cette base interdisciplinaire a façonné une approche holistique, positionnant l’activité ciblée comme un moteur de la santé et du bien-être. L’identité épistémique de l’ergothérapeute était fondée sur la sagesse pratique – une compréhension profonde, basée sur l’expérience, de la guérison par l’action. Même à ses débuts, la profession devait équilibrer ce qui était observable, ce qui était ressenti et ce qui était théorisé.

Au fil du temps, en particulier en réponse à l’impact de la guerre, notre profession a évolué (Dillingham, 2002; Eldar & Jelić, 2003). Après les deux guerres mondiales, elle est devenue étroitement liée à la réadaptation des militaires blessés, recourant aux activités de la vie quotidienne pour restaurer à la fois la fonction physique et la résilience psychologique (Eldar & Jelić, 2003). Les premières théories de la réadaptation et les observations empiriques ont façonné la pratique, fusionnant les connaissances tacites et les cadres scientifiques émergents. Dans les années 1940, les connaissances en ergothérapie ont commencé à être de plus en plus fondées sur la recherche et la rigueur scientifique (Singal, 1987). Les progrès en matière de neuroplasticité et d’apprentissage moteur ont élargi le domaine, tandis que l’accent croissant mis sur la recherche a cherché à mettre l’ergothérapie en phase avec les paradigmes médicaux et scientifiques (Stockmeyer, 1967). Dans les années 1970, les organismes de recherche et les revues professionnelles ont renforcé les approches positivistes de la connaissance, priorisant les résultats objectifs et mesurables par rapport aux formes de compréhension tacites, intuitives ou relationnelles (Mosey, 1970; Mosey, 1985).

### L’identité épistémique en 2025

En 2025, l’ergothérapie est bien ancrée dans les secteurs de la santé et des services sociaux, témoignant d’une riche histoire qui reflète une tension continue entre rigueur scientifique et art de la pratique. La profession s’est élargie en matière de portée, de technologie et de cadres fondés sur des données probantes; cependant, des tensions épistémiques fondamentales persistent. Bien que la pratique fondée sur les données probantes ait renforcé la crédibilité clinique de l’ergothérapie, certains membres de la communauté de recherche mettent en garde contre le fait qu’une trop grande importance accordée aux résultats mesurables pourrait amener à négliger des éléments humanistes cruciaux, tels que l’empathie, l’intuition, l’art de la guérison, et l’importance du contexte et de l’expérience clinique (Hinojosa, 2013; Reid et al., 2024; Whiteford, 2005). Bien que plus difficiles à quantifier, ces qualités restent vitales pour une pratique thérapeutique efficace.

La jeune mère dans une communauté éloignée n’a pas seulement besoin d’interventions appuyées par la recherche; elle a besoin de solutions qui s’adaptent au désordre de la vie réelle. L’ergothérapeute doit intégrer l’intuition, la créativité et l’expertise clinique aux données probantes pour offrir des soins efficaces et significatifs. Les connaissances *scientifiques et empiriques* éclairent ses décisions cliniques; les stratégies de protection des articulations, les cadres de réadaptation et le suivi des symptômes aident à façonner son plan de traitement. Des *connaissances fondées sur la pratique*, développées grâce à des années d’expérience, lui permettent d’anticiper les défis, de prioriser les soins dans un contexte où le temps est limité, et de déterminer quand faire confiance à son intuition plutôt qu’à des protocoles stricts (Higgs & Titchen, 2001; Kinsella, 2012). Les *connaissances de la clientèle* sont centrales; l’expérience vécue par la mère, les stratégies de gestion de la douleur et les réalités de son environnement éloigné dictent ce qui sera efficace pour elle (Dumez & L’Espérance, 2024). *La connaissance esthétique* nourrit la créativité, l’empathie et le lien émotionnel, rappelant à l’ergothérapeute que la guérison englobe non seulement les dimensions physiques, mais aussi les dimensions psychologiques, émotionnelles et spirituelles (Gorodeisky & Marcus, 2022).

### Défendre et définir notre vision pluraliste du savoir

L’ergothérapie se nourrit du pluralisme épistémique (Zangwill, 2020), soit la capacité de tisser ensemble ces diverses formes de savoirs dans des soins significatifs et sensibles au contexte (Kinsella, 2012). Cette diversité est une force. Elle permet aux ergothérapeutes de faire face aux complexités de l’occupation humaine avec adaptabilité et discipline intellectuelle. Cependant, le pluralisme ne signifie pas que toutes les connaissances ont un poids égal dans tous les contextes. Sans critères d’intégration clairs, nous risquons de franchir une ligne, de l’inclusion réfléchie au relativisme épistémique, où toute perspective est considérée comme valide, quel que soit son fondement.

Nous devons constamment faire face à des tensions critiques : comment pouvons-nous nous assurer que les ergothérapeutes qui s’appuient sur l’intuition fondent tout de même leurs décisions sur des données rigoureuses? Lorsque les récits des personnes patientes divergent des résultats empiriques, comment naviguer parmi ces perspectives et les concilier? Et comment se prémunir contre la désinformation ou contre l’anecdote qui affaiblit notre base de connaissances plutôt que de l’enrichir?

Pendant des décennies, l’ergothérapie a cherché à concilier avec de multiples modes de connaissance, mais la façon dont nous justifions et appliquons ces connaissances a évolué. Sans intégration intentionnelle, nous risquons de nous fragmenter, ou pire, de permettre aux responsables politiques, aux bailleurs de fonds et aux professions adjacentes de définir ce qui constitue une connaissance légitime en ergothérapie. La solution n’est pas une hiérarchisation rigide où une forme de connaissance domine, mais une interaction structurée qui garantit que nos savoirs restent inclusifs, défendables et pratiques. Notre force ne réside pas dans le rejet du savoir empirique, mais dans son rapprochement rigoureux et intentionnel avec la sagesse issue de la pratique, la perspicacité relationnelle et l’expérience vécue. Les fausses dichotomies – science versus expérience, résultats mesurables versus participation significative – nous freinent. Nous n’avons pas à choisir; nous devons intégrer. Le véritable défi n’est pas seulement de reconnaître la pluralité des savoirs, mais de les mobiliser intentionnellement et de les inscrire dans les systèmes qui façonnent les pratiques et les politiques en santé. Comment alors assurer que cette approche pluraliste, mais rigoureuse, soit non seulement reconnue, mais soutenue dans la recherche, la pratique et les politiques? C’est là que les communautés épistémiques deviennent essentielles.

Plus que de simples réseaux de partage des connaissances, les communautés épistémiques définissent ce qui constitue la légitimité (Haas, 1992). Elles la valident et la mobilisent, intégrant divers modes de connaissance dans des systèmes d’influence. En garantissant que le savoir reste adapté aux contextes sociaux et politiques, elles plaident pour qu’il soit intégré dans les systèmes de soins de santé de manière rigoureuse et reconnue.

## Section 2 : Le rôle des ergothérapeutes dans les communautés épistémiques : contributions aux politiques et à la pratique

### Introduction aux communautés épistémiques

Les communautés épistémiques jouent depuis longtemps un rôle crucial dans la résolution de défis mondiaux complexes. Par exemple, les collaborations entre biologistes, économistes de l’environnement et spécialistes des sciences climatiques ont façonné des politiques internationales importantes, telles que le Protocole de Kyoto (Nations, 1998) et l’Accord de Paris (Nations, 2016). De même, l’apport des réseaux d’épidémiologistes, de spécialistes de la santé publique et de plusieurs professions prestataires de soins de santé a été crucial pour coordonner les réponses de l’Organisation mondiale de la santé aux pandémies telles que le VIH/SIDA, le SRAS et la COVID-19 (Mediterranean, 2024). Ces communautés intègrent adroitement les points de vue de divers domaines, dont les sciences fondamentales, les sciences sociales, le droit et l’économie, afin de créer des stratégies nuancées qui réduisent les inégalités, favorisent des politiques inclusives et stimulent le changement systémique.

À la fin du XX^e^ siècle, le sociologue P. M. Haas a introduit le concept de communautés épistémiques comme « réseaux de professionnels ayant une expertise reconnue dans un domaine particulier et revendiquant une autorité en ce qui concerne les connaissances pertinentes pour les politiques publiques » (Haas, 1992, p. 3).

#### L’appartenance à une communauté épistémique est-elle une question d’expertise, de reconnaissance ou de pouvoir?

Cross (MAIA’K, 2013) soutient que les communautés épistémiques ne sont pas simplement l’intégralité d’une profession, et qu’elles ne sont pas non plus limitées à ses membres. Bien que ces communautés soient souvent liées à des scientifiques, elles comprennent aussi des personnes qui détiennent une expertise hors du milieu scientifique. Ces groupes peuvent être composés de professionnelles et professionnels divers, dont des analystes des politiques, des porte-parole et d’autres personnes détentrices de connaissances clés, pas seulement dans les domaines scientifiques ou techniques. Fait important, les communautés épistémiques sont légitimes parce que leurs membres sont considérés comme crédibles dans un domaine particulier et peuvent parvenir à un consensus sur des questions essentielles. Cependant, leur influence n’est pas inhérente à leur statut; elle est façonnée par l’évolution de la dynamique du pouvoir et des facteurs politiques qui déterminent comment leurs contributions sont reconnues et mises en œuvre dans l’élaboration des politiques (Haas, 1992).

### Les critères clés d’une communauté épistémique

Pour que les communautés épistémiques fonctionnent efficacement, elles doivent répondre à quatre critères clés – exprimés par sept modes de fonctionnement – qui permettent une influence sur les politiques et une collaboration significative vers des objectifs communs (Haas, 1992; Smirnova & Yachin, 2015).

1. *Un ensemble partagé de valeurs et de convictions fondées sur des normes et des principes :* Un trait clé d’une communauté épistémique est son engagement à l’égard de valeurs et de principes directeurs partagés. Les *convictions fondées sur des normes* unissent les membres autour d’objectifs communs et de principes éthiques, comme l’engagement des ergothérapeutes envers une réadaptation équitable et centrée sur les clientèle. *Les convictions fondées sur des principes* façonnent la façon dont l’information est comprise et appliquée, comme l’hypothèse selon laquelle la pratique fondée sur des données probantes améliore les résultats pour la clientèle. La communauté fonctionne selon deux principes centraux : *un engagement commun à l’égard de la vérité*, reconnaissant que la vérité peut être comprise de différentes manières; une priorité accordée aux connaissances scientifiques crédibles par rapport aux intérêts personnels ou politiques; et une *adhésion aux valeurs partagées*, assurant une approche cohérente et axée sur les valeurs de la résolution de problèmes et de la défense des droits et intérêts en matière de politiques publiques.
2. *Des convictions partagées quant aux causes ou une compréhension partagée :* La deuxième caractéristique est la compréhension partagée par la communauté des causes profondes derrière les défis qu’elle cherche à relever. Cela permet aux membres de créer des interventions ciblées et des solutions politiques et est opérationnalisé de deux façons : par la *collaboration interdisciplinaire*, où par exemple, des personnes ayant une expertise en ergothérapie, en santé publique, en droit et en éducation collaborent pour améliorer l’accès aux services qui optimisent la participation et réduisent les disparités en matière de santé, et par l’*influence politique*, où un engagement collectif à l’égard d’une question mobilise le soutien, façonne les politiques et favorise le changement systémique.
3. *Une définition commune de la validité :* Une troisième caractéristique est une compréhension partagée des connaissances valides et fiables. Les normes communes guident les membres dans l’évaluation des données probantes, la résolution des problèmes et la formulation de recommandations en matière de politiques. Ce critère se manifeste de deux façons : par la *responsabilité personnelle –* chaque membre est responsable de fournir des données probantes rigoureuses et une expertise professionnelle pour éclairer la prise de décision – et par l’*adhésion à des critères communs concernant les connaissances valides –* assurer un consensus sur ce qui constitue des connaissances fiables, qu’elles soient basées sur la recherche scientifique, les expériences vécues ou les meilleures pratiques cliniques –, en maintenant la cohérence dans la façon dont les données probantes sont évaluées et appliquées.
4. *Un engagement partagé à l’égard de l’action – une entreprise politique commune :* Le dernier trait est centré sur une mission commune consistant à relever les défis par le biais d’une expertise collective. Les membres collaborent pour créer un impact tangible, en utilisant leur savoir pour améliorer le bien-être et stimuler le changement. Les principaux modes de fonctionnement comprennent un *accent sur les enjeux globaux*, garantissant que les efforts sont consacrés à des problèmes systémiques et à long terme affectant le développement humain et le bien-être de la société, et l’*auto-organisation*, qui permet aux membres de travailler de manière autonome dans leurs domaines de spécialisation tout en collaborant stratégiquement pour produire des solutions transformatrices.

### L’ergothérapie COMME communauté épistémique

Les ergothérapeutes génèrent, valident et diffusent activement des connaissances, un processus dans lequel nous nous engageons quotidiennement. À ce titre, l’ergothérapie fonctionne *comme* une communauté épistémique : une communauté épistémique *interne* qui renforce la légitimité et l’avancement de notre profession. Voici trois exemples de la façon dont nous fonctionnons en tant que communauté épistémique :

- *Réseaux de recherche :* Grâce à des collaborations nationales et internationales, les ergothérapeutes contribuent à la production de données probantes en utilisant diverses approches. Ces approches comprennent des études qui utilisent des essais contrôlés randomisés ainsi que des méthodes de recherche plus participatives et inclusives. De telles collaborations améliorent notre compréhension des déterminants de l’occupation humaine et nous permettent de développer et de tester des interventions qui améliorent les résultats pour la clientèle.
- *Associations professionnelles :* Des organisations comme l’Association canadienne des ergothérapeutes (CAOT, n.d.) et la Fédération mondiale des ergothérapeutes (WFOT, n.d.) favorisent le partage des connaissances par le biais de conférences, d’énoncés de position et de lignes directrices cliniques qui influent sur la profession.
- *Cadres pédagogiques :* Dans les milieux universitaires, les programmes d’ergothérapie préparent la communauté étudiante à évaluer de manière critique et à intégrer les données probantes dans divers environnements de pratique, en veillant à ce que les personnes diplômées puissent fournir des soins de haute qualité et que la profession continue d’évoluer sur la base de connaissances solides (ACOTUP, n.d.).

#### L’ergothérapie DANS LE CADRE d’un réseau épistémique interdisciplinaire plus large

Les ergothérapeutes jouent également un rôle essentiel dans les communautés épistémiques interdisciplinaires plus larges. Tout comme les climatologues et les responsables politiques qui unissent leurs forces pour relever les défis environnementaux et de santé publique, les ergothérapeutes travaillent aux côtés des responsables politiques, des membres de la communauté d’ingénierie, des autres professionnels de santé, des gestionnaires, des économistes, des responsables de la santé publique, des spécialistes des sciences sociales et de la population.

Par l’entremise de la collaboration interdisciplinaire, de la défense des droits et intérêts et de la mobilisation des connaissances, les ergothérapeutes façonnent activement les politiques, l’éducation et la pratique en matière de soins de santé afin d’améliorer l’accès à la réadaptation et de favoriser le changement systémique. Nous offrons une contribution épistémique unique à ces grandes communautés en y incorporant nos perspectives fondées sur l’occupation ainsi que nos approches axées sur les personnes patientes, et en mettant l’accent sur la participation comme résultat en matière de santé. Cela représente une *communauté épistémique externe.* Voici un exemple :

Je fais partie d’une communauté épistémique vouée à l’avancement des soins de réadaptation axés sur la personne et fondés sur des données probantes pour des millions de personnes au Canada qui en ont besoin. Ayant travaillé à rapprocher les perspectives de l’éducation, de la pratique et des politiques pour promouvoir des pratiques fondées sur les données probantes, je reconnaissais qu’il en fallait davantage. Pour renforcer ces liens, plusieurs collègues et moi-même avons travaillé en collaboration pour explorer comment nous pouvions être plus efficaces. Au fil du temps, nous avons créé une communauté diversifiée de membres de la communauté de recherche dans les domaines des services de santé, de la réadaptation, de la science de la mise en œuvre, de la sociologie, de l’anthropologie, des sciences politiques, de l’économie de la santé, de la géographie et de l’épidémiologie. Nous avons collaboré avec des parties prenantes de l’industrie, des établissements d’enseignement, des porte-parole des personnes patientes, des associations professionnelles, des organismes de réglementation, des hôpitaux de réadaptation, des organismes communautaires, des responsables politiques, des représentantes et représentants du gouvernement et des partenaires et communautés autochtones. Notre communauté comprend également des prestataires de soins de santé travaillant en première ligne, comme des membres de la communauté d’ergothérapie, de physiothérapie, de chiropractie, des soins infirmiers, de médecine, et de psychologie, qui travaillent en soins primaires, en réadaptation et en milieu communautaire. En quoi sommes-nous une communauté épistémique?

*Des convictions normatives partagées :* Nous sommes unis par un engagement fondamental à l’égard de soins de réadaptation axés sur la personne et fondés sur des données probantes. Nous croyons que l’accès rapide à des services de réadaptation de qualité est un droit, et non un privilège, et que la collaboration interdisciplinaire est essentielle pour y parvenir. Ces valeurs communes guident nos efforts pour combler les écarts entre la recherche, les politiques et la pratique.

*Des convictions partagées quant aux causes :* Nous reconnaissons que les obstacles entravant l’accès à la réadaptation vont au-delà des facteurs cliniques et sont étroitement liés aux cadres politiques, économiques et sociaux. Pour surmonter ces obstacles, il faut adopter une approche multisectorielle intégrant les connaissances tirées de la recherche sur les services de santé, de la science de la mise en œuvre, de la sociologie et des politiques, tout en impliquant activement les partenaires autochtones, les porte-parole des clientèles, les leaders de l’industrie et les représentants et représentantes du gouvernement. En intégrant ces points de vue, nous visons à élaborer des solutions fondées sur des données probantes et pertinentes au contexte qui améliorent l’accès à la réadaptation ainsi que les résultats partout au Canada.

*Une définition commune de la validité :* Nous défendons une production de connaissances rigoureuse et interdisciplinaire qui valorise les données probantes qualitatives et quantitatives, ainsi que les différences contextuelles et les expériences vécues. Nous reconnaissons que le succès ne se mesure pas uniquement par les essais cliniques, mais aussi par l’impact réel, l’engagement communautaire et les approches tenant compte des différences culturelles.

*Une entreprise politique commune :* Nous nous engageons à élaborer des modèles de soins durables qui garantissent un accès rapide et équitable à la réadaptation. Notre mission en matière de politiques publiques favorise l’intégration de la réadaptation dans les soins primaires et communautaires, renforce les initiatives de santé des Autochtones et donne la priorité à la réadaptation dans les politiques de soins de santé. En collaborant avec les responsables politiques, les organismes de réglementation et les associations professionnelles, nous visons à façonner l’avenir de la réadaptation au Canada.

Aujourd’hui, notre communauté épistémique comprend près de 60 membres dévoués qui, surtout au cours des deux dernières années, ont travaillé sans relâche vers un objectif commun : s’assurer que les personnes reçoivent des services de réadaptation lorsqu’elles en ont le plus besoin, au bon moment, et auprès des prestataires de soins de santé appropriés.

### Du concept à l’action : pourquoi cela est important pour l’avenir de l’ergothérapie

Les communautés épistémiques ne sont pas seulement des réseaux abstraits : elles façonnent l’identité professionnelle, influencent les politiques de santé et entraînent des changements tangibles dans le monde réel. L’ergothérapie se trouve à un carrefour : nous pouvons façonner activement notre rôle dans l’évolution des systèmes de santé, ou nous laisser définir par les autres. Trois forces sont à l’origine de cette urgence : 1) au niveau de la profession; 2) au niveau des connaissances; 3) au niveau des systèmes :

Au niveau de la profession : Le paysage des soins de santé évolue, et l’ergothérapie doit clairement définir son rôle; sinon, celui-ci risque d’être défini par les autres. Alors que les modèles de soins se transforment, que les structures de financement changent et que la privatisation prend de l’ampleur, notre profession ne peut pas se permettre de rester passive. Par exemple, une occasion clé réside dans l’intégration des soins primaires, où l’expertise des ergothérapeutes en soins préventifs et communautaires est inestimable. Cependant, être valorisés ne suffit pas; nous devons affirmer de manière proactive notre place au sein de ces modèles. Les professions qui se positionnent stratégiquement dans des secteurs en pleine croissance gagnent en influence dans le financement, l’élaboration des politiques et la conception des systèmes. Si nous ne défendons pas clairement nos droits et intérêts, notre profession risque de se voir marginalisée ou absorbée dans des disciplines plus larges, ce qui minerait ses contributions distinctes. Pendant ce temps, nos débats internes, comme les distinctions entre généralistes et spécialistes (Foto, 1996), ne doivent pas nous diviser. Au lieu de les considérer comme un problème, nous devrions en tirer parti pour clarifier et renforcer notre position dans le domaine des soins de santé. La politique de santé privilégie les professions organisées et stratégiques par rapport aux approches fragmentées.

Au niveau des connaissances : Le partage interdisciplinaire des connaissances est essentiel en ergothérapie; cependant, les silos professionnels, les contraintes réglementaires et les définitions rigides des données probantes entravent souvent la collaboration. Prenons, par exemple, la pandémie de COVID-19, qui a nécessité des innovations rapides, accélérant l’adoption de la télésanté et nécessitant une collaboration interdisciplinaire pour maintenir les soins (Omboni et al., 2022; Sharma et al., 2022; Shaver, 2022). Au départ, les ergothérapeutes ont dû faire face à des obstacles – langages professionnels, cadres réglementaires et modèles de financement divergents –, en particulier dans le contexte de la réadaptation après un AVC, où la coordination est cruciale (Harahsheh et al., 2022; Hubert et al., 2021; Kimmel et al., 2024). Néanmoins, ces défis ont également favorisé l’innovation, menant à une meilleure intégration des systèmes, à la mise à jour des lignes directrices en matière de télésanté et à une collaboration accrue. Les ergothérapeutes ont joué un rôle central dans l’adaptation de la prestation des services, le renforcement des réseaux et l’intégration de la réadaptation dans les modèles de soins virtuels. Malgré ces contributions, les connaissances issues de l’ergothérapie ont souvent été écartées des décisions politiques, révélant un enjeu plus profond : *Qu’est-ce qui constitue une connaissance valide?*

La pratique traditionnelle fondée sur les données probantes donne la priorité aux essais cliniques, assure la rigueur et minimise les préjugés (Library, 2025). Cependant, l’expérience vécue, la recherche qualitative et les approches participatives fournissent des informations tout aussi vitales (Baum et al., 2006; Cargo & Mercer, 2008; Greenhalgh & Papoutsi, 2018). La pandémie a mis en évidence les limites des cadres de pratique étroits fondés sur les données probantes; bien que l’efficacité de la télésanté ait été confirmée, les évaluations ont rarement tenu compte de l’accessibilité, des besoins de réadaptation à long terme ou des soins tenant compte des différences culturelles. Si l’ergothérapie vise à influencer la transformation des soins de santé, nous devons remettre en question les définitions dépassées des données probantes, plaider pour des systèmes de connaissances plus inclusifs et veiller à ce que notre expertise façonne le changement à l’échelle du système au lieu de se contenter d’y réagir.

Au niveau des systèmes : Les soins de santé évoluent – le vieillissement des populations, la mondialisation, les pénuries de main-d’œuvre et les contraintes en matière de ressources redéfinissent la prestation des services (Elmslie, 2012; Kolb, 2018; Wong, 2014). La COVID-19 a accéléré ces changements, mettant en évidence à la fois les vulnérabilités du système et les possibilités pour l’ergothérapie de prendre les devants en matière d’innovation, de renforcer les soins axés sur les personnes patientes et d’influencer les politiques (Nana-Sinkam et al., 2021). Au fur et à mesure que l’espérance de vie augmente, la demande de modèles de réadaptation durables augmente aussi (Lindgren, 2016; Meinow et al., 2022; Organization, 2017). L’IA transforme les soins de santé, mais l’ergothérapie doit veiller à ce que la technologie ne remplace pas, mais améliore les soins centrés sur l’être humain, préservant les relations thérapeutiques et l’expertise clinique.

L’ergothérapie démontre son plein potentiel à l’intersection des soins cliniques, de l’éducation, et à l’élaboration des politiques et de la recherche. Cependant, les silos structurels peuvent entraver la mise en œuvre des meilleures pratiques (Amri et al., 2022). La question de la réadaptation après un AVC illustre cet enjeu : alors que les données probantes appuient les modèles de rétablissement complets et axés sur la personne patiente, les obstacles politiques entravent souvent leur adoption dans le monde réel (Baatiema et al., 2017; Cormican et al., 2023). Les ergothérapeutes doivent collaborer avec le personnel administratif des hôpitaux, les responsables politiques et les bailleurs de fonds pour positionner la réadaptation comme un investissement, plutôt que comme un aspect auquel on pense après coup. En comblant les écarts entre les soins de santé, l’éducation et les services sociaux, l’ergothérapie peut entraîner des changements à l’échelle du système et renforcer son rôle dans le façonnement de l’avenir des soins de santé.

### Pour la suite des choses : la force de l’intégration

Notre force réside dans l’intégration de diverses formes de connaissances – la science, la pratique, l’expérience vécue et la sagesse communautaire. Cependant, l’ergothérapie englobe plus que des connaissances; elle met l’accent sur la connexion – rapprocher les disciplines, traduire les idées en actions et promouvoir la participation. Ces atouts nous permettent de relever les défis mondiaux. Nous sommes à la fois une communauté professionnelle distincte et une partie essentielle de réseaux interdisciplinaires plus larges. Ce double rôle peut être notre force, mais seulement si nous l’acceptons. Le clarifier nous permet de définir nous-mêmes notre identité au lieu de laisser les autres le faire pour nous. Plutôt que de remettre en question notre place, nous devons affirmer notre leadership en matière de santé, de bien-être et de justice.

## Section 3 : Appels à l’action pour renforcer le rôle et l’impact des ergothérapeutes dans les communautés épistémiques

L’ergothérapie se trouve à la croisée des chemins – et il faut le voir non pas comme une épreuve à traverser, mais bien comme une occasion de faire preuve de leadership. Les valeurs fondamentales qui sont promues aujourd’hui dans le domaine de la santé – soins personnalisés, reconnaissance holistique des individus, pratique axée sur les relations et données probantes de haute qualité – sont ancrées dans notre profession depuis longtemps. Pour que l’ergothérapie et la réadaptation restent à l’avant-garde des soins de santé, nous devons passer de la reconnaissance à l’action : partager notre expertise, adopter divers modes de connaissance, plaider pour des politiques transformatrices et amorcer des conversations plus larges sur les soins de santé. Nous avons déjà une place dans ces espaces. Le moment est venu d’aller de l’avant avec détermination et leadership.

Dans la suite de ce texte, je lance trois appels à l’action pour renforcer notre base de connaissances, amplifier notre voix collective et accroître notre influence dans le façonnement de l’avenir de la profession, de la réadaptation et des soins de santé au sens large.

### Appel n° 1 : Renforcer notre base de connaissances : faire preuve d’humilité épistémique, élargir la notion de données probantes et combler les lacunes entre la recherche et la pratique

#### Faire preuve d’humilité épistémique

Au cœur d’une communauté épistémique se trouve un engagement envers le respect mutuel, la collaboration et l’humilité épistémique – la reconnaissance du fait que la connaissance n’est pas uniquement basée sur l’expertise individuelle, mais est façonnée par la sagesse collective (Kidd, 2017; Maduro, 2012). Il s’agit de valoriser les voix traditionnellement exclues de la planification et de la prestation des soins et d’intégrer les connaissances issues de la santé publique, de l’urbanisme, de l’économie, de la médecine intégrative, de l’ingénierie, de la sociologie et de la géographie, entre autres. Ces partenariats remettent en question nos présupposés et élargissent notre compréhension de phénomènes importants.

Comment cultiver cette humilité? Je propose un cadre en six étapes axé sur l’action pour intégrer des connaissances diversifiées dans la formation, la pratique et les politiques en ergothérapie. Ce processus doit commencer tôt et demeurer au cœur de notre profession, ce qui nécessite un engagement structuré dans les programmes de formation et les milieux de stage, et des discussions professionnelles entre le personnel enseignant, le personnel clinique, les gestionnaires et la clientèle :

- Écouter (bien entendre). Une écoute authentique exige de l’ouverture, de la patience et une volonté d’accueillir des perspectives qui remettent en question les nôtres.
- Tolérer (laisser de l’espace). Le dialogue constructif laisse de l’espace au désaccord, sans forcer le consensus.
- Réfléchir (s’arrêter et évaluer). Il faut évaluer de manière critique comment le savoir est construit, qui est inclus, et quels sont les présupposés à l’œuvre.
- Apprendre (grandir). Le savoir est fluide et évolutif; il n’est pas fixe ou certain. On grandit lorsqu’on embrasse cette réalité.
- Tenter (essayer avec courage). Le changement nécessite des actions. Il faut prendre des risques, expérimenter de nouveaux modèles et considérer l’échec comme faisant partie intégrante du processus d’apprentissage.
- Évaluer (réétalonner). Évaluer, c’est une occasion d’apprendre, pas seulement de mesurer le succès. Il faut comprendre ce qui fonctionne, ce qui ne fonctionne pas, et pourquoi.

#### Élargir la notion de données probantes et combler les lacunes entre la recherche et la pratique

Les ergothérapeutes jouent un rôle essentiel dans la création, le partage et l’application du savoir sous toutes ses formes. Nous devons nous engager à chaque étape, en veillant à ce que les divers systèmes de connaissances soient reconnus et que les nouvelles connaissances reflètent les expériences vécues des clientèle. Comment pouvons-nous y parvenir dans le contexte de nos communautés épistémiques?

1. *Création du savoir :* Nous devons produire un savoir qui saisit les complexités de l’(in)capacité, de la fonction et de la participation par l’entremise de méthodes participatives, ancrées dans la pratique, et de cocréation. Cela nécessite une innovation sur le plan méthodologique, des recherches menées par la communauté et des données probantes du monde réel pour assurer la pertinence et l’inclusivité. La création du savoir doit englober des cadres non occidentaux et intégrer diverses perspectives épistémiques dans la recherche sur la réadaptation. Cela implique de mobiliser les leaders de la santé autochtones, de codévelopper des approches communautaires et de tirer des enseignements des systèmes de santé ailleurs dans le monde.
2. *Diffusion du savoir :* Le savoir doit atteindre les bons publics de manière à promouvoir l’action. Nous devons déterminer non seulement qui le reçoit, mais aussi comment il est communiqué et appliqué. Une diffusion efficace garantit que les données probantes sortent du monde universitaire pour avoir un impact sur la pratique et les politiques.
3. *Mise en œuvre du savoir :* La mise en œuvre n’est pas facultative, elle est au cœur de notre rôle. Nous devons soutenir activement l’adoption du savoir que nous générons, en veillant à ce qu’il se traduise en des changements significatifs dans les pratiques et les politiques.

Deux questions critiques influencent les trois étapes : 1) *Reconnaître les structures de pouvoir* – combler le fossé entre la recherche et la pratique nécessite de déterminer qui contrôle le savoir et de qui l’expertise est valorisée (Kitto et al., 2012; Rycroft-Malone et al., 2016). Nous devons remettre en question ces hiérarchies pour créer des systèmes de connaissances inclusifs et axés sur les personnes patientes; et 2) *Promouvoir l’engagement critique et la réflexivité* – le savoir évolue par la réflexion critique, le dialogue et l’adaptation (Ng et al., 2022; Sherwood, 2024). L’intégration de la réflexivité dans la formation, la recherche et la pratique nous permet d’examiner de manière critique nos présupposés, nos valeurs et notre positionnalité par rapport aux soins. Cela améliore notre capacité à remettre en question les préjugés, à répondre aux besoins émergents et à défendre une réadaptation éthique, socialement juste et axée sur la clientèle.

*Appel à la prudence!* Élargir notre compréhension du savoir ne signifie pas abandonner la pratique fondée sur les données probantes. Bien que celle-ci puisse parfois marginaliser les connaissances expérientielles ou tacites, elle demeure une protection essentielle contre les préjugés et la désinformation. Nous devons élargir notre définition des données probantes tout en reconnaissant les limites des approches empiriques traditionnelles. Si les *données probantes* continuent de servir de norme pour la légitimité, nous devons articuler cette réalité, reconnaître ses contraintes et aller de l’avant sans nous excuser – non pas pour remplacer une approche par une autre, mais pour les réconcilier.

### Appel n° 2 : Renforcer notre voix collective : clarifier notre identité, réduire la fragmentation, façonner la perception du public

#### Clarifier notre identité, réduire la fragmentation et façonner la perception du public

Si nous sommes fractionnés à l’interne, nous minons notre communauté épistémique et notre capacité à défendre efficacement nos droits et intérêts dans les communautés interdisciplinaires. Notre adaptabilité est une force, mais notre influence collective diminue si nous n’énonçons pas clairement nos rôles, notre portée et nos contributions. Pour amplifier notre impact, nous devons nous unir autour d’une vision commune – une vision qui intègre la recherche, la pratique et les principes de justice sociale qui sous-tendent notre travail. L’unité, cependant, ne nécessite pas une normalisation rigide; elle implique plutôt des principes partagés, des compétences fondamentales et une appréciation des divers contextes. En cocréant des principes directeurs qui définissent notre impact, notre expertise et nos interventions, nous renforçons notre influence sur les politiques, le financement et l’innovation. Cette initiative stratégique nous permet de collaborer entre les disciplines et d’accroître notre impact dans les domaines de la santé mentale, des soins en matière de démence, des technologies de réadaptation, et de l’inclusion communautaire des personnes ayant des incapacités. Voici où nous devons concentrer nos efforts :

1. *Tirer parti des différences pour l’innovation :* Les disparités régionales, la confusion des rôles et le cloisonnement des domaines de pratique affaiblissent notre influence. Cependant, ces différences peuvent aussi constituer des avantages stratégiques. Plutôt que d’éviter les conversations difficiles, nous devons analyser les causes profondes de la fragmentation, faciliter les débats structurés pour combler les lacunes, et transformer la diversité des points de vue en solutions novatrices pour la pratique, la politique et la formation. La cohésion n’est pas synonyme d’uniformité; il s’agit d’aligner stratégiquement notre expertise pour faire avancer la profession.
2. *Établir des espaces de dialogue permanents :* Le changement n’est pas le fruit d’une seule conversation. Pour influencer les politiques et les stratégies, nous avons besoin de forums structurés et soutenus. Cela implique la création d’espaces de discussion au sein des associations professionnelles, des forums interdisciplinaires et de l’arène des politiques nationales, la promotion de la collaboration interdisciplinaire et la participation active des ergothérapeutes aux discussions sur les politiques nationales. Si notre profession n’est pas incluse dans la conversation, elle sera écartée des décisions qui façonnent l’avenir des soins de santé.
3. *Clarifier ce qui constitue notre valeur unique, renforcer la défense de nos droits et intérêts :* Qu’est-ce qui distingue l’ergothérapie des autres professions? Pour renforcer notre influence, nous devons clairement définir et communiquer nos compétences de base, axer notre défense des droits et intérêts sur les priorités clés en matière de soins de santé, et veiller à ce que notre message soit cohérent, persuasif et fondé sur des données. En affinant la façon dont nous transmettons notre valeur, nous renforçons notre statut professionnel tout en maintenant notre caractère distinctif.

### Appel n° 3 : Renforcer notre influence : collaboration, impact sur les politiques et leadership

Les ergothérapeutes ont l’expertise nécessaire pour influencer les politiques de santé, mais la profession demeure insuffisamment reconnue. Sans efforts ciblés en matière de défense des droits et intérêts, elle risque d’être laissée à l’écart des décisions importantes en matière de soins de santé. Notre rôle va au-delà du travail clinique : nous aidons à établir des priorités, à élaborer des interventions et à gérer les ressources. Le changement de système nécessite que l’ergothérapie soit présente dans les cercles décisionnels, pas qu’elle reste sur la touche. Ce changement doit commencer tôt, en intégrant l’engagement politique et les compétences en défense des droits et des intérêts dans l’enseignement de l’ergothérapie, et ce, dès le début.

#### La collaboration comme catalyseur d’influence

La collaboration dans le domaine des soins de santé n’est pas facultative; elle est essentielle. L’ergothérapie, par sa nature même, est intrinsèquement collaborative, nécessitant un engagement avec la clientèle, les médecins, le personnel des soins de longue durée et d’autres professionnels et professionnelles. Elle se trouve à l’intersection des soins, reliant les disciplines et assurant la continuité entre les phases de traitement. Les ergothérapeutes doivent aller au-delà des bonnes intentions en définissant et en communiquant activement leurs contributions uniques tout en favorisant des partenariats soutenus dont l’impact est mesurable à l’échelle locale, nationale et internationale. Le renforcement de ces collaborations jette les bases d’un changement politique significatif. Voici comment nous pouvons le mettre en pratique :

*Engagement interdisciplinaire pour l’intégration du système :* Pour nous assurer un rôle dans le façonnement des soins de santé, nous devons nous engager dans une variété de disciplines et de secteurs. Cela implique par exemple de :

- Coorganiser des ateliers avec les administrations d’hôpitaux et les équipes de réadaptation pour améliorer les soins aux personnes ayant subi un AVC.
- Participer à des tables rondes sur l’innovation dans les soins de santé pour faire valoir l’impact de l’ergothérapie sur la réduction des réadmissions à l’hôpital.
- Établir des comités permanents avec les prestataires de soins primaires afin d’améliorer l’aiguillage précoce pour les affections musculosquelettiques.

*Partenariats institutionnels pour un impact durable :* L’atteinte d’une influence durable nécessite une collaboration structurelle avec les organisations clés.

- Élaborer en collaboration un cadre national pour la prestation de services de réadaptation avec l’Association canadienne des ergothérapeutes.
- Intégrer du contenu sur l’ergothérapie dans les programmes de formation interdisciplinaires en santé à l’université pour s’assurer que les futurs professionnels prestataires des soins de santé l’apprécient.
- Établir un partenariat avec des groupes de défense des clientèles, comme le Réseau des patients partenaires, afin de cocréer des ressources axées sur l’accès à la réadaptation.

*Réseaux mondiaux pour une portée plus large :* L’ergothérapie s’étend au-delà des frontières nationales. Voici des exemples :

- Travailler en partenariat avec l’OMS sur des initiatives pour vieillir en santé.
- Contribuer aux directives de la Fédération mondiale des ergothérapeutes (FME) en matière de réadaptation.
- S’engager au sein de consortiums de recherche mondiaux, tels que la Global Alliance for Rehabilitation and Assistive Technology (Headquarters, 2019).

Bien que la collaboration soit essentielle pour accroître notre impact, influencer les politiques nécessite un changement stratégique dans la façon dont nous intégrons la réadaptation dans les systèmes de soins de santé.

### De l’influence au leadership : accroître notre influence sur les politiques

Les ergothérapeutes doivent sortir de la marge en repensant la réadaptation comme une priorité de santé clé et en s’attaquant au préjugé en faveur des soins actifs qui la marginalise souvent. Nous devons faire de la réadaptation un élément essentiel à la viabilité du système de santé et plaider pour son intégration dans les politiques.

*Intégrer l’ergothérapie dans les politiques :* Les ergothérapeutes n’ont pas besoin d’être des spécialistes en matière de politiques pour réaliser des changements. En participant aux cadres clés et aux décisions de financement, nous pouvons nous assurer que la réadaptation est reconnue et dotée de ressources adéquates. Cela signifie : plaider pour un financement à la hauteur du rôle de la réadaptation dans la prévention et les soins de longue durée, participer aux discussions politiques nationales et internationales, comme l’initiative *Réadaptation 2030* de l’OMS (Organization, 2025), pour intégrer l’ergothérapie dans les réformes des soins de santé, mais aussi, tirer parti de la recherche en économie et en politiques publiques pour démontrer la rentabilité de l’ergothérapie en matière de soins primaires, de santé mentale et de vieillissement.

*Élargir la définition des données probantes dans l’élaboration des politiques :* Les décisions politiques donnent souvent la priorité aux essais cliniques et aux indicateurs biomédicaux; cependant, la réadaptation doit également intégrer les récits des personnes patientes et leurs expériences vécues, ainsi que les interventions portées par la communauté qui saisissent les répercussions réelles, de même que la recherche sur le système de santé démontrant les avantages pratiques de la réadaptation.

*Rapprocher recherche et politiques pour un impact en temps réel :* Combler l’écart entre la recherche et la pratique exige de solides mécanismes de partage des connaissances. Les ergothérapeutes doivent aider à traduire la recherche sur la réadaptation en recommandations politiques concrètes, à engager directement les responsables politiques pour s’assurer que la prise de décision repose sur des données probantes et à mettre en place des réseaux intersectoriels pour positionner l’ergothérapie comme un élément central de la transformation des soins de santé. En combinant la collaboration stratégique et l’engagement actif en matière de politiques, les ergothérapeutes peuvent passer du statut de simples participants à celui de maîtres d’œuvre de l’avenir des politiques de réadaptation.

## Conclusion

### Incarner le savoir que nous détenons : de l’identité à l’action

L’ergothérapie évolue depuis toujours pour répondre aux besoins de la société – du traitement moral à la réadaptation en temps de guerre, en passant par la pratique fondée sur les données probantes et le recours aux technologies de pointe. Ce qui a commencé comme un concept radical – l’idée selon laquelle l’activité signifiante favorise la guérison – s’est transformé en une discipline fondée sur la science, la sagesse clinique et l’expérience personnelle. Alors que les soins de santé deviennent de plus en plus complexes, nous n’avons pas besoin de certitudes absolues, mais de clarté. Nous ne sommes pas seulement des prestataires de services, nous sommes des gestionnaires de connaissances, des influenceuses et influenceurs politiques et des personnes influentes de la justice occupationnelle. Cependant, au fil de notre progression, nous devons nous poser la question suivante : Est-ce que nous évoluons de manière intentionnelle, ou est-ce que nous nous contentons de réagir?

L’ergothérapie est plus qu’une simple profession; elle forme une communauté épistémique unifiée par une expertise partagée et un dévouement à comprendre comment l’occupation affecte le bien-être. Cependant, nos convictions collectives sont insuffisantes à elles seules. Les défis à venir – iniquités en matière de santé, modèles de soins en évolution et prise de décision fondée sur l’IA – exigent une profession cohérente, confiante et adaptable. Si nous ne parvenons pas à affirmer quelle est notre place, d’autres la délimiteront pour nous. Notre rôle ne devrait pas se limiter à des tâches techniques. Il devrait plutôt être lié au savoir humaniste et contextuel qui renforce notre efficacité.

Pourquoi devrions-nous être optimistes quant au pouvoir de notre communauté de savoir? Parce que je nous ai vus en action.

*Dans le milieu de l’enseignement supérieur :* Nous remettons en question les programmes d’études en ergothérapie, exposons leur curriculum caché, et faisons évoluer les méthodes d’enseignement et d’évaluation. Nous faisons le lien entre le milieu universitaire et la pratique grâce à un engagement plus profond avec la communauté étudiante, les sites cliniques et les précepteurs et préceptrices.

*Dans la pratique :* Nous travaillons en partenariat avec les communautés autochtones, les ONG et les organisations communautaires pour cocréer des soins tenant compte des différences culturelles. Nous menons des initiatives sur le vieillissement, la santé des enfants et la santé mentale, en nous assurant que les personnes y participent selon leurs propres conditions. Nous avons un impact – non seulement en nous intéressant aux résultats concernant les incapacités, mais aussi en encourageant la participation et l’engagement occupationnels.

*Dans les politiques publiques :* Nous mobilisons les décisionnaires et travaillons avec l’ONU, la FME et l’OMS. Si nous frappions autrefois aux portes des responsables politiques, ceux-ci frappent désormais aux nôtres.

Je vous laisse avec les questions suivantes – qui doivent être vues non pas comme des sujets de réflexion abstraits, mais comme des impératifs : Que pouvons-nous faire pour mieux soutenir la prochaine génération d’ergothérapeutes dans l’acquisition de connaissances et de compétences critiques, réflexives et adaptatives? Comment pouvons-nous renforcer notre impact collectif en renforçant notre implication dans la collaboration interdisciplinaire dans les domaines des soins de santé, de l’éducation et des politiques publiques? Comment pouvons-nous adopter une approche plus proactive pour façonner les discussions sur les politiques entourant la réadaptation, la participation et l’équité en matière de santé, en tirant parti des moments significatifs dans la société? Quels engagements prendrons-nous, individuellement et collectivement, pour incarner en action les valeurs d’une communauté épistémique?

*Aller de l’avant en toute confiance :* Notre profession s’épanouit en trouvant un équilibre entre structure et flexibilité, entre tradition et innovation, ainsi qu’entre science et art. Ce n’est pas une faiblesse; cela représente notre plus grande force. Cela nous permet de nous adapter tout en nous appuyant sur des principes fondamentaux. Nous valorisons le pluralisme, l’humilité, la collaboration, la réflexivité et le courage de s’engager dans un désaccord constructif. Nous devons avancer avec la confiance, la vision et la force collective de notre profession. Le savoir que nous détenons est puissant, mais seulement si nous le mobilisons ensemble. En embrassant notre identité épistémique complète, nous renforçons notre profession, améliorons la participation et la qualité de vie et promouvons la justice et l’équité entre les individus, les communautés et les systèmes entiers.

## Remerciements

Merci à l’Association canadienne des ergothérapeutes pour cet honneur extraordinaire. Je suis reconnaissante à Lori Letts, Bernadette Nedelec et Margo Paterson d’avoir proposé ma candidature et pour leur appui et leurs encouragements indéfectibles. Merci à mes collègues et camarades de l’École de physiothérapie et d’ergothérapie de l’Université McGill, dont la perspicacité et la générosité m’ont guidée à chaque étape de mon parcours. Merci également à mes étudiantes et étudiants des cycles supérieurs : j’ai appris autant de vous que vous avez appris de moi. Enfin, à ma famille — Harry, Zoé et Nicholas — merci d’être ma boussole, ma joie et la force qui rend tout possible.

## References

[bibr1-00084174251383259a] ACOTUP (n.d.). Homepage. https://www.acotup-acpue.ca/English/.

[bibr2-00084174251383259a] AlcoffL. M. (2010). Epistemic identities. Episteme, 7(2), 128–137.

[bibr3-00084174251383259a] AmriM. ChaturA. O’CampoP. (2022). An umbrella review of intersectoral and multisectoral approaches to health policy. Social Science & Medicine, 315, 115469.36403353 10.1016/j.socscimed.2022.115469

[bibr4-00084174251383259a] AndersenL. ReedK. (2024). The history of occupational therapy: The first century. Taylor & Francis.

[bibr5-00084174251383259a] BaatiemaL. OtimM. E. MnatzaganianG. de-Graft AikinsA. CoombesJ. SomersetS. (2017). Health professionals’ views on the barriers and enablers to evidence-based practice for acute stroke care: a systematic review. Implementation Science, 12, 1–15.28583164 10.1186/s13012-017-0599-3PMC5460544

[bibr6-00084174251383259a] BaumF. MacDougallC. SmithD. (2006). Participatory action research. Journal of epidemiology and community health, 60(10), 854.16973531 10.1136/jech.2004.028662PMC2566051

[bibr7-00084174251383259a] CAOT (n.d.). Homepage. http://www.caot.ca.

[bibr8-00084174251383259a] CargoM. MercerS. L. (2008). The value and challenges of participatory research: strengthening its practice. Annu. Rev. Public Health, 29(1), 325–350.18173388 10.1146/annurev.publhealth.29.091307.083824

[bibr9-00084174251383259a] CormicanA. HiraniS. P. McKeownE. (2023). Healthcare professionals’ perceived barriers and facilitators of implementing clinical practice guidelines for stroke rehabilitation: A systematic review. Clinical rehabilitation, 37(5), 701–712.36475911 10.1177/02692155221141036PMC10041573

[bibr10-00084174251383259a] DillinghamT. R. (2002). Physiatry, physical medicine, and rehabilitation: historical development and military roles. Physical Medicine and Rehabilitation Clinics, 13(1), 1–16.11878077 10.1016/s1047-9651(03)00069-x

[bibr11-00084174251383259a] DumezV. L’EspéranceA. (2024). Beyond experiential knowledge: a classification of patient knowledge. Social Theory & Health, 22(3), 173–186.

[bibr12-00084174251383259a] DuntonW. R. (1919). Reconstruction therapy . WB Saunders Company.

[bibr13-00084174251383259a] EldarR. JelićM. (2003). The association of rehabilitation and war. Disability and rehabilitation, 25(18), 1019–1023.12944156 10.1080/0963828031000137739

[bibr14-00084174251383259a] ElmslieK . (2012). Against the growing burden of disease. *Ottawa: Public Health Agency of Canada*, Centre for Chronic Disease Prevention.

[bibr15-00084174251383259a] to, M (1996). Generalist versus specialist occupational therapists. The American Journal of Occupational Therapy: Official Publication of the American Occupational Therapy Association, 50(10), 771–774.8947368 10.5014/ajot.50.10.771

[bibr16-00084174251383259a] FrickerM. (2007). Epistemic Injustice: Power and the Ethics of Knowing. Oxford University Press. 10.1093/acprof:oso/9780198237907.001.0001 .

[bibr17-00084174251383259a] GorodeiskyK. MarcusE. (2022). Aesthetic knowledge. Philosophical studies, 179(8), 2507–2535.

[bibr18-00084174251383259a] GreenhalghT. PapoutsiC. (2018). Studying complexity in health services research: desperately seeking an overdue paradigm shift. In, 16, 1–6. Springer.10.1186/s12916-018-1089-4PMC600905429921272

[bibr19-00084174251383259a] HaasP. M. (1992). Introduction: epistemic communities and international policy coordination. International organization, 46(1), 1–35.

[bibr20-00084174251383259a] HarahshehE. EnglishS. W. HrdlickaC. M. DemaerschalkB. (2022). Telestroke’s role through the COVID-19 pandemic and beyond. Current Treatment Options in Neurology, 24(11), 589–603.35999901 10.1007/s11940-022-00737-0PMC9388966

[bibr21-00084174251383259a] HeadquartersW. (2019). Global perspectives on assistive technology.

[bibr22-00084174251383259a] HiggsJ. TitchenA. (2001). Rethinking the practice-knowledge interface in an uncertain world: A model for practice development. British Journal of Occupational Therapy, 64(11), 526–533.

[bibr23-00084174251383259a] HinojosaJ. (2013). The evidence-based paradox. The American Journal of Occupational Therapy, 67(2), e18–e23.23433283 10.5014/ajot.2013.005587

[bibr24-00084174251383259a] HornA. UriasE. KleinJ. T. HessA. ZweekhorstM. B. (2023). Expert and non-expert at the same time: knowledge integration processes and dynamics in interdisciplinary teamwork. Sustainability Science, 18(5), 2357–2371.

[bibr25-00084174251383259a] HubertG. J. CoreaF. SchlachetzkiF. (2021). The role of telemedicine in acute stroke treatment in times of pandemic. Current Opinion in Neurology, 34(1), 22–26.33230037 10.1097/WCO.0000000000000887

[bibr26-00084174251383259a] KiddI. J. (2017). Confidence, humility, and hubris in Victorian scientific naturalism. Epistemic virtues in the sciences and the humanities, 11–25.

[bibr27-00084174251383259a] KimmelB. WalderA. OvalleA. Venkatasubba RaoC. P. SavitzS. I. GoldbergM. WarachS. Cruz-FloresS. OlsonD. M. AndersonJ. A. (2024). Stroke survivors’ telemedicine experiences during the COVID-19 pandemic: A phenomenological investigation. Digital Health, 10, 20552076241281464.39381819 10.1177/20552076241281464PMC11459570

[bibr28-00084174251383259a] KinsellaE. A. (2012). Knowledge paradigms in occupational science: Pluralistic perspectives. Occupational science: Society, inclusion, participation, 67–85.

[bibr29-00084174251383259a] KittoS. C. SargeantJ. ReevesS. SilverI. (2012). Towards a sociology of knowledge translation: the importance of being dis-interested in knowledge translation. Advances in Health Sciences Education, 17, 289–299.21638085 10.1007/s10459-011-9303-6

[bibr30-00084174251383259a] KolbM. (2018). What is globalization? And how has the global economy shaped the United States. *Peterson Institute for International Economics*. https://www. *piie*. *com/microsites/globalization/what-is-globalization*.

[bibr31-00084174251383259a] LibraryU. o. C. (2025). *Hierarchy of Evidence – Evidence-Based Practice in Health*. Evidence-Based Practice in Health (LibGuides). https://canberra.libguides.com/c.php?g=599346&p=4149721.

[bibr32-00084174251383259a] LindgrenB. (2016). The rise in life expectancy, health trends among the elderly, and the demand for care-a selected literature review.

[bibr33-00084174251383259a] LyallC. BruceA. TaitJ. MeagherL. (2011). Interdisciplinary research journeys: Practical strategies for capturing creativity. Bloomsbury Academic.

[bibr34-00084174251383259a] MaduroO. (2012). An (other) invitation to epistemological humility: Notes toward a self-critical approach to counter-knowledges. Decolonizing epistemologies: Latina/o theology and philosophy, 87–103.

[bibr35-00084174251383259a] MAI’A K, D. C (2013). Rethinking epistemic communities twenty years later. Review of international studies, 39(1), 137–160.

[bibr36-00084174251383259a] Mediterranean, W. H. O. R. O. f. t. E. (2024). *Outbreaks – Epidemic and pandemic-prone diseases*. https://www.emro.who.int/pandemic-epidemic-diseases/outbreaks/index.html.

[bibr37-00084174251383259a] MeinowB. LiP. JasilionisD. OksuzyanA. SundbergL. KelfveS. WastessonJ. W. (2022). Trends over two decades in life expectancy with complex health problems among older Swedes: implications for the provision of integrated health care and social care. BMC Public Health, 22(1), 759.35421981 10.1186/s12889-022-13099-8PMC9012017

[bibr38-00084174251383259a] MoseyA. C. (1970). Three Frames of Reference for Mental Health. C. B. Slack. https://books.google.ca/books?id=LwC7AAAAIAAJ .

[bibr39-00084174251383259a] MoseyA. C. (1985). Eleanor Clarke Slagle lecture, 1985: a monistic or a pluralistic approach to professional identity? The American Journal of Occupational Therapy, 39(8), 504–509.4037048 10.5014/ajot.39.8.504

[bibr40-00084174251383259a] Nana-SinkamP. KraschnewskiJ. SaccoR. ChavezJ. FouadM. GalT. AuYoungM. NamoosA. WinnR. SheppardV. (2021). Health disparities and equity in the era of COVID-19. Journal of clinical and translational science, 5(1), e99.34192054 10.1017/cts.2021.23PMC8167251

[bibr41-00084174251383259a] NationsU. (1998). Kyoto protocol to the united nations framework convention on climate change.

[bibr42-00084174251383259a] NationsU. (2016). *The Paris Agreement*. (FCCC/CP/2015/10/Add.1). United Nations Framework Convention on Climate Change (UNFCCC).

[bibr43-00084174251383259a] NgS. L. ForseyJ. BoydV. A. FriesenF. LangloisS. LadonnaK. MylopoulosM. SteenhofN. (2022). Combining adaptive expertise and (critically) reflective practice to support the development of knowledge, skill, and society. Advances in Health Sciences Education, 27(5), 1265–1281.36350488 10.1007/s10459-022-10178-8PMC9645329

[bibr44-00084174251383259a] OmboniS. PadwalR. S. AlessaT. BenczúrB. GreenB. B. HubbardI. KarioK. KhanN. A. KonradiA. LoganA. G. LuY. MarsM. McManusR. J. MelvilleS. NeumannC. L. ParatiG. RennaN. F. RyvlinP. SanerH. WangJ. (2022). The worldwide impact of telemedicine during COVID-19: current evidence and recommendations for the future. Connect Health, 1, 7–35. 10.20517/ch.2021.0335233563 PMC7612439

[bibr45-00084174251383259a] OrganizationW. H . (2017). Rehabilitation: key for health in the 21st century (WHO/NMH/NVI/17.2). H. i. s. a. r. W. H. Organization.

[bibr46-00084174251383259a] OrganizationW. H . (2025). *Rehabilitation 2030 initiative*. https://www.who.int/initiatives/rehabilitation-2030

[bibr47-00084174251383259a] ReidH. HockingC. SmytheE. (2024). Occupational therapy’s oversight: How science veiled our humanity. Scandinavian Journal of Occupational Therapy, 31(1), 2306585.38261715 10.1080/11038128.2024.2306585

[bibr48-00084174251383259a] Rycroft-MaloneJ. BurtonC. R. BucknallT. GrahamI. D. HutchinsonA. M. StaceyD. (2016). Collaboration and co-production of knowledge in healthcare: opportunities and challenges. International journal of health policy and management, 5(4), 221.27239867 10.15171/ijhpm.2016.08PMC4818986

[bibr49-00084174251383259a] SharmaA. PruthiM. SageenaG. (2022). Adoption of telehealth technologies: an approach to improving healthcare system. Translational medicine communications, 7(1), 20.35967767 10.1186/s41231-022-00125-5PMC9361246

[bibr50-00084174251383259a] ShaverJ. (2022). The State of Telehealth Before and After the COVID-19 Pandemic. Prim Care, 49(4), 517–530. 10.1016/j.pop.2022.04.00236357058 PMC9035352

[bibr51-00084174251383259a] SherwoodG. (2024). Reflective practice and knowledge development: Transforming research for a practice-based discipline. In (Vol. 11, pp. 399-404): Elsevier.

[bibr52-00084174251383259a] SingalD. J. (1987). Towards a definition of American modernism. American Quarterly, 39(1), 7–26.

[bibr53-00084174251383259a] SmirnovaM. Y. YachinS. Y. (2015). Epistemic communities and epistemic operating mode. International Journal of Social Science and Humanity, 5(7), 646.

[bibr54-00084174251383259a] StockmeyerS. A. (1967). An interpretation of the approach of Rood to the treatment of neuromuscular dysfunction. American journal of physical medicine & rehabilitation, 46(1), 900–956.6023297

[bibr55-00084174251383259a] WFOT (n.d.). Homepage. https://wfot.org/.

[bibr56-00084174251383259a] WhitefordG. (2005). Knowledge, power, evidence: A critical analysis of key issues in evidence based practice. In Occupation & practice in context (pp. 34–50). Elsevier.

[bibr57-00084174251383259a] WongA. (2014). Government of Canada—Action for Seniors Report. *Government of Canada* .

[bibr58-00084174251383259a] ZangwillN. (2020). Epistemic pluralism: The missing link and the ambitions of epistemology. Metaphilosophy, 51(4), 485–498.
